# Multi-Omics and Functional Analysis of BFSP1 as a Prognostic and Therapeutic Target in Liver Hepatocellular Carcinoma

**DOI:** 10.3390/medicina61122196

**Published:** 2025-12-11

**Authors:** Kyu-Shik Lee, Jongwan Kim

**Affiliations:** 1 Department of Pharmacology, College of Medicine, Dongguk University, Gyeongju 38066, Republic of Korea; there1@dongguk.ac.kr; 2 Department of Anatomy, College of Medicine, Dongguk University, Gyeongju 38066, Republic of Korea

**Keywords:** prognostic biomarker, tumor-infiltrating immune cell, DNA methylation, bioinformatics analysis

## Abstract

*Background and Objectives:* Although beaded filament structural protein 1 (BFSP1) may be involved in oncogenic mechanisms, its clinical relevance and functional role in liver hepatocellular carcinoma (LIHC) remain unclear. This study examined the prognostic significance, regulatory mechanisms, and potential therapeutic implications of BFSP1 in LIHC. *Materials and Methods:* Comprehensive bioinformatics analysis was performed across multiple platforms using datasets derived from The Cancer Genome Atlas. Differential gene expression, DNA methylation, copy number variation, immune cell infiltration, drug sensitivity, and co-expression networks were systematically examined. Functional enrichment analyses of protein–protein and gene–gene interaction networks were conducted using STRING and GeneMANIA. Additionally, short interfering RNA-mediated knockdown and wound-healing assays were performed in HepG2 cells to evaluate BFSP1 function in vitro. *Results:* The results showed that BFSP1 mRNA expression was significantly upregulated in tissues from LIHC patients. Elevated BFSP1 levels were associated with poorer prognostic patterns, which were further supported by detailed clinicopathological subgroup analyses. Furthermore, BFSP1 expression was correlated with promoter hypomethylation and associated with patterns of tumor-infiltrating immune cells, including specific immune cell subtypes such as M1 and M2 macrophages. Integrative analyses revealed strong associations between BFSP1 and drug sensitivity, as well as a regulatory network encompassing genes involved in the cell cycle, DNA repair, and metabolic processes. Functional knockdown of BFSP1 significantly reduced HepG2 cell migration in vitro, as assessed by wound healing assay, with decreased wound closure at 24 h (11.0% vs. 16.5%) and 48 h (7.4% vs. 12.5%) compared with the control (*p* < 0.05, *n* = 6 biological replicates). *Conclusions:* In conclusion, these findings suggest that BFSP1 functions as a multifaceted prognostic biomarker and a potential therapeutic target for LIHC.

## 1. Introduction

Liver hepatocellular carcinoma (LIHC), the predominant histological subtype of primary liver cancer, presents a major global health challenge. It accounts for over 90% of all primary liver malignancies and consistently ranks among the leading causes of cancer-related mortality worldwide, occupying the third, fourth, or sixth position depending on the region and data source [1,2,3,4]. The disease disproportionately affects individuals with underlying chronic liver conditions, as 80–90% of cases arise in the context of cirrhosis. Among patients with cirrhosis, the annual incidence of LIHC is estimated at 2% to 4% [5]. Established etiological factors in LIHC include chronic infection with hepatitis B or C viruses, alcohol-related liver disease, non-alcoholic fatty liver disease, and exposure to metabolic or environmental carcinogens [6,7].

Despite extensive research efforts and notable advancements in surgical, locoregional, and systemic therapies, the prognosis of patients with LIHC remains poor, with a 5-year survival rate below 20% and an overall survival (OS) among the lowest of all solid tumors [7,8,9]. These unfavorable outcomes are largely attributable to late-stage diagnosis, substantial tumor heterogeneity, high recurrence rates, and a pronounced tendency for early metastasis [10,11]. Moreover, LIHC is frequently diagnosed at an advanced stage, which precludes curative treatment and leads to poor survival outcomes.

Traditional prognostic models that rely on clinical and pathological parameters are insufficient to capture the molecular and biological complexity of LIHC, thereby constraining their utility in guiding individualized patient management [12]. Comprehensive molecular profiling encompassing genomic, epigenomic, and transcriptomic analyses can facilitate identification of robust prognostic and predictive biomarkers, enabling the development of precise oncology approaches [13,14,15]. Recent high-throughput studies have significantly advanced the understanding of the molecular landscape of LIHC, as well as the critical roles of genetic mutations, epigenetic modifications, and immune microenvironmental factors in tumor initiation, progression, and therapeutic response. The identification and validation of novel biomarkers with both prognostic and therapeutic relevance are critically important.

Beaded filament structural protein 1 (BFSP1), also known as filensin, is a type VI intermediate filament protein initially characterized as a critical structural component of the ocular lens, where it contributes to maintaining lens transparency and fiber cell integrity [16,17,18]. BFSP1 assembles specialized intermediate filaments in conjunction with BFSP2 and is closely related to aquaporin 0, another membrane protein specific to the lens [16]. Although traditionally considered as an eye lens-specific cytoskeletal protein, recent pan-cancer analyses have identified aberrant expression of BFSP1 in various malignancies, including LIHC, suggesting its role in oncogenic processes beyond ocular tissues [19,20].

Emerging evidence indicates that BFSP1 functions as an independent risk factor and adverse prognostic biomarker in liver cancer [21]. Several studies reported that BFSP1 expression is significantly upregulated in LIHC tissues and cell lines compared with normal controls, with higher BFSP1 levels correlating with poor clinical outcomes [19,21]. Furthermore, BFSP1 has been implicated in epigenetic and post-transcriptional regulatory mechanisms, including N6-methyladenosine mRNA methylation, which is closely associated with patient survival and tumor progression in LIHC [19,22]. Additionally, BFSP1 may be involved in carbohydrate metabolic pathways, highlighting its pleiotropic role in cancer biology [22]. However, the mechanisms and clinical implications of BFSP1 dysregulation in LIHC remain poorly understood.

The immune microenvironment exerts a significant influence on tumorigenesis, disease progression, and therapeutic response in LIHC [23,24,25]. Tumor-infiltrating immune cells (TIICs), which encompass diverse lymphoid and myeloid populations, play critical roles in modulating tumor behavior and shaping host antitumor immunity [26,27]. The density, phenotype, and spatial distribution of TIICs are closely associated with patient prognosis and have emerged as important determinants of clinical outcomes and the effectiveness of immunotherapies in liver cancer [28,29]. Notably, systemic immune-based therapies, including immune checkpoint inhibitors, have demonstrated promising efficacy in patients with advanced LIHC, particularly those who are not candidates for surgical resection or liver transplantation [30]. Traditionally, prognostic evaluation and therapeutic decision-making in liver cancer have predominantly depended on tumor burden, hepatic function, and patient performance status [31]. Nevertheless, the clinical relevance of tumor biomarkers has gained increasing recognition, and risk models incorporating molecular and immune parameters are now being utilized to predict survival, metastasis, and recurrence in patients with LIHC [32,33]. Integrating tumor biomarker profiles with immune microenvironment features into predictive models may enhance risk stratification, optimize treatment selection, and ultimately improve patient outcomes.

Aberrant DNA methylation is a hallmark of cancer and plays a critical role in LIHC initiation and progression [2,34,35,36,37]. Promoter hypermethylation results in epigenetic silencing of tumor suppressor genes, whereas promoter hypomethylation can lead to activation of oncogenes, which collectively contribute to tumorigenesis and malignant transformation [35,36,38,39]. Importantly, these characteristic DNA methylation changes often occur in the early stages of carcinogenesis, highlighting their potential utility as biomarkers for early cancer detection [40,41,42,43]. In LIHC, both genome-wide and locus-specific methylation alterations have been documented, and DNA methylation profiles have been demonstrated to correlate with prognosis, therapeutic response, and disease recurrence [44,45,46,47,48,49]. In addition to alterations within tumor tissues, transcriptomic and epigenetic modifications in adjacent nontumoral cirrhotic tissues are associated with clinical outcomes, demonstrating the complexity of the epigenetic landscape in LIHC [50]. Methylation signatures derived from both tumor and non-tumor tissues exhibit prognostic significance, particularly in patients with early-stage tumors undergoing curative resection [51]. Although numerous studies have explored DNA methylation alterations in LIHC [44,52,53,54,55], investigations into the dynamic epigenetic changes occurring during the progression from cirrhosis or preneoplastic lesions to overt carcinoma are limited. Moreover, dysregulation of DNA methylation enzymes such as DNA methyltransferases has been implicated in the pathogenesis of hepatitis B virus-related LIHC by contributing to silencing of tumor suppressor genes and aberrant activation of oncogenic pathways [56,57]. Despite substantial progress in understanding the role of DNA methylation in LIHC, further research is necessary to characterize the temporal and spatial dynamics of methylation changes and to validate DNA methylation-based biomarkers for clinical applications.

Copy number variations (CNVs), defined as DNA segments exceeding 1 kb that differ from the reference genome, are important structural genetic alterations in human cancers [57,58]. CNVs include both duplications and deletions of chromosomal regions, induction of deviations from the normal diploid state, and contribution to genomic instability [59,60,61]. These variations can lead to activation of oncogenes or inactivation of tumor suppressor genes, thereby promoting tumorigenesis and facilitating malignant transformation [58,62]. Furthermore, CNVs can modulate gene expression which impacts key cancer hallmarks such as uncontrolled proliferation, metastatic potential, drug resistance, and immune evasion [63,64,65]. Aneuploidy, defined as an abnormal chromosome number, is a prevalent type of CNV and a critical driver of tumorigenesis across various cancer types [66,67]. The extent and distribution of both broad and focal CNVs are differentially correlated with gene expression patterns, particularly those related to cellular proliferation and immune regulation [63,65]. Notably, the impact of CNVs on tumor behavior and the immune microenvironment is highly context-dependent and may vary substantially depending on cancer types [68,69,70].

LIHC is intrinsically resistant to conventional chemotherapeutic agents, with most patients demonstrating limited sensitivity to standard drug regimens and a marked propensity to develop multidrug resistance during therapy [71,72]. These features markedly diminish the effectiveness of systemic treatment, resulting in poor clinical outcomes and reduced survival rates. However, pharmacotherapy remains a fundamental component in the management of advanced LIHC and other malignancies, as it can prolong survival and improve the quality of life of selected patients [73,74]. Therefore, drug sensitivity testing is a promising strategy for guiding personalized chemotherapy for specific cancer subtypes. Nevertheless, therapeutic efficacy is often compromised by the emergence of resistance to antineoplastic agents, which is driven by both genetic alterations (e.g., mutations, CNVs) and non-genetic mechanisms (e.g., epigenetic modifications, tumor microenvironmental factors) [75,76,77,78]. Accumulating clinical evidence highlights the pivotal role of these molecular determinants in mediating both intrinsic and acquired drug resistance in LIHC, underscoring the urgent need to comprehensively understand the genetic and epigenetic landscapes influencing therapeutic responses. Determining the mechanisms underlying drug resistance and identifying reliable predictive biomarkers are essential for optimizing individualized treatment strategies and improving the clinical outcomes of patients with LIHC.

## 2. Materials and Methods

### 2.1. mRNA Expression Analysis of BFSP1 in LIHC

To systematically investigate the mRNA expression profile of BFSP1 in LIHC, a comprehensive integrative bioinformatics approach utilizing multiple publicly available databases was employed. Specifically, Tumor Immune Estimation Resource (TIMER) 2.0, Gene Set Cancer Analysis (GSCA), and UALCAN were applied for multidimensional transcriptomic analyses, each providing distinct analytical frameworks and datasets primarily derived from The Cancer Genome Atlas (TCGA) [79,80,81]. Initially, differential expression of BFSP1 between LIHC tumor tissues and corresponding normal controls was assessed using TIMER2.0 (http://timer.cistrome.org/; accessed on 7 April 2025), which offers robust, standardized gene expression estimates across pan-cancer cohorts and supports rigorous tumor versus normal comparisons based on preprocessed TCGA RNA-seq data [2,80,81,82]. Subsequently, the GSCA platform (https://guolab.wchscu.cn/GSCA/#/; accessed on 7 April 2025) was used to integrate the mRNA expression, DNA methylation, immune infiltration metrics, and survival data from TCGA and additional large-scale cohorts. This step enabled multifaceted evaluation of BFSP1 expression in LIHC, including analysis of the relationships between BFSP1 expression, methylation status, immune cell infiltration, and clinical outcomes [83]. Finally, UALCAN (http://ualcan.path.uab.edu/; accessed on 8 April 2025), a user-friendly TCGA-based web portal, was used to examine BFSP1 expression in various clinicopathological subgroups of LIHC. UALCAN facilitates subgroup analyses based on sample type (tumor versus normal), primary tumor, tumor stage, tumor grade, histological subtype, and *TP53* mutation to precisely determine expression patterns within clinically relevant strata [2,79,82,84].

### 2.2. Prognostic Analysis of BFSP1 in LIHC

To evaluate the prognostic value of BFSP1 in LIHC, comprehensive survival analyses were conducted using several publicly accessible bioinformatics platforms, including the Kaplan–Meier plotter (http://kmplot.com/analysis/; accessed on 9 April 2025) [2,82], GSCA, OSlihc (https://bioinfo.henu.edu.cn/LIHC/LIHCList.jsp; accessed on 9 April 2025) [82], and PrognoScan (http://dna00.bio.kyutech.ac.jp/PrognoScan/; accessed on 9 April 2025) [85,86]. Kaplan–Meier survival curves were generated to compare outcomes between patient subgroups stratified by high versus low BFSP1 expression, employing platform-specific automated or median-based cut-off thresholds. The assessed survival endpoints included OS, progression-free interval, disease-free interval, and disease-specific survival (DSS). Hazard ratios (HRs) and 95% confidence intervals (CIs) were calculated using log-rank test and Cox proportional hazard regression models implemented within each platform. Because cut-off values are defined automatically within these tools and cannot be harmonized across platforms, our interpretation emphasized both statistical significance and the consistency and magnitude of the effect sizes (HRs) across multiple analyses to ensure a robust assessment of prognostic relevance. Notably, these analyses utilized the comprehensive sample size of the TCGA-LIHC cohort integrated into these platforms, ensuring adequate statistical power for subgroup comparison based on the established prognostic cut-off thresholds (such as the median), thereby minimizing the risk of underpowering.

### 2.3. DNA Methylation and Prognostic Analysis of BFSP1 in LIHC

To investigate the DNA methylation status of BFSP1 and its prognostic significance in LIHC, several publicly accessible bioinformatics platforms were utilized, including OncoDB (https://oncodb.org/; accessed on 12 April 2025) [2], UALCAN, MethSurv (https://biit.cs.ut.ee/methsurv/; accessed on 12 April 2025), Shiny Methylation Analysis Resource Tool [SMART; (http://www.bioinfo-zs.com/smartapp/; accessed on 12 April 2025)], and MEXPRESS (https://mexpress.be/; accessed on 12 April 2025) [2,87,88,89,90]. Initially, the methylation profiles of BFSP1 were examined using OncoDB, which provides comprehensive DNA methylation data derived from TCGA, enabling direct comparison of methylation values between normal liver and LIHC tissues. Subsequently, UALCAN was employed for subgroup-specific analyses, facilitating the evaluation of BFSP1 promoter methylation in relation to clinicopathological parameters, such as tumor stage and grade. The SMART platform was used to validate correlations between methylation and gene expression of BFSP1 across multiple cancer types, which enabled visualization of the inverse relationship between gene expression and DNA methylation, which aided in identification of potential epigenetic regulatory mechanisms. For site-specific methylation analysis, MEXPRESS was used to map CpG island methylation across the BFSP1 locus and correlate these data with gene expression and clinical features. MEXPRESS integrates expression, methylation, and clinical annotations from TCGA-LIHC, displaying CpG site-specific methylation levels along with genomic structure and transcription start sites; however, because the platform does not allow user-defined control over the multiple testing correction procedures, our ability to uniformly manage false discovery rate adjustments across all CpG sites was inherently limited. Similarly, to assess the prognostic relevance of BFSP1 methylation, MethSurv was used to conduct site-specific methylation-survival analyses based on TCGA-LIHC data, generating Kaplan–Meier survival curves for individual CpG sites within BFSP1. As with MEXPRESS, MethSurv applies its built-in statistical pipeline, and therefore, the multiple testing correction for the large number of CpG site could not be standardized beyond the platform’s default implementation.

### 2.4. Immune Infiltration and Drug Sensitivity Analysis of BFSP1 in LIHC

To investigate the association between BFSP1 expression and the tumor immune microenvironment in LIHC, immune infiltration analyses were performed using the GSCA platform (https://guolab.wchscu.cn/GSCA/#/; accessed on 13 April 2025). This platform incorporates pre-calculated immune cell infiltration scores derived from TCGA-LIHC RNA-sequencing data [89,91]. Spearman’s correlation analysis, as implemented in GSCA, was used to assess the relationships between BFSP1 expression and the abundance of various immune cell types, including B cells, CD4+ T cells, CD8+ T cells, macrophages, neutrophils, and dendritic cells. To control for false-positive findings, multiple-testing correction was applied using FDR, with statistically significant defined as FDR < 0.05. Additionally, drug sensitivity analyses were conducted by examining the correlations between gene expression and drug response using data from the Cancer Therapeutics Response Portal (CTRP) and Genomics of Drug Sensitivity in Cancer (GDSC) databases, both integrated within the GSCA platform, with FDR-adjusted *p*-values used to determine statistical significance.

### 2.5. Gene–Chemical Interaction Analysis of BFSP1

To explore the potential interactions between BFSP1 and various chemical compounds, the Comparative Toxicogenomics Database [CTD; (http://ctdbase.org/; accessed on 14 April 2025)] was used. CTD is a manually curated resource that integrates data linking gene expression profiles to environmental chemical exposure and pharmacological agents [92]. Documented associations between BFSP1 and various chemicals, including pharmaceuticals and environmental toxicants, were identified and catalogued to provide insight into the potential modulators of BFSP1 expression and possible mechanisms by which chemical exposure affects BFSP1-related pathways in LIHC.

### 2.6. Co-Expression Network and Functional Enrichment Analysis of BFSP1

To explore the co-expression network and functional enrichment of BFSP1 in LIHC, large-scale integrative omics analysis was performed using the LinkedOmics platform (http://www.linkedomics.org; accessed on 15 April 2025), which is based on TCGA datasets to enable large-scale integrative omics analysis [82,93]. The “LinkFinder” module within LinkedOmics was used to identify genes that were significantly co-expressed with BFSP1 in TCGA-LIHC cohort by computing Pearson’s correlation coefficients. For functional annotation, Gene Set Enrichment Analysis (GSEA) was performed on BFSP1-associated gene sets using the “LinkInterpreter” module of LinkedOmics. Enrichment analyses of Gene Ontology (GO) terms and Kyoto Encyclopedia of Genes and Genomes (KEGG) pathways were performed, with statistical significance determined through 500 permutations and a FDR threshold of less than 0.05. Enriched GO terms and KEGG pathways were reported based on the default ranking and visualization tools provided by the LinkedOmics platform.

### 2.7. Prognostic Analysis of Co-Expressed Genes Associated with BFSP1 in LIHC

To assess the prognostic significance of genes co-expressed with BFSP1 in LIHC, the Gene Expression Profiling Interactive Analysis (GEPIA) 2 platform (http://gepia2.cancer-pku.cn; accessed on 15 April 2025) was utilized [82,94]. Genes found to be significantly co-expressed with BFSP1 through LinkedOmics analysis were input into the GEPIA2 “Survival Map” module to evaluate their associations with patient survival outcomes.

### 2.8. Gene–Gene Interaction Network Analysis of BFSP1 in LIHC

To determine the gene–gene interaction network associated with BFSP1 in LIHC, the GeneMANIA platform (http://www.genemania.org; accessed on 16 April 2025) was used [95]. GeneMANIA constructs comprehensive interaction networks by integrating multiple data types, including physical protein–protein interactions, gene co-expression, co-localization, pathway associations, and genetic interactions. BFSP1 was designated as the query gene, and a gene–gene interaction network was generated to identify potential interactors and functionally related genes.

### 2.9. Protein–Protein Interaction (PPI) Network and Enrichment of BFSP1

For Protein–Protein Interaction (PPI) network analysis of BFSP1 in LIHC, PPI networks were constructed using the STRING database (https://string-db.org; accessed on 17 April 2025), which integrates experimentally validated and computationally predicted protein interactions from diverse sources [96]. BFSP1 was queried using STRING, and a PPI network was generated using a medium confidence interaction score to identify both direct and indirect protein partners. GO and KEGG pathway enrichment analyses were conducted on the BFSP1-centered PPI network using the functional enrichment tools embedded in STRING. Enriched biological processes, molecular functions, cellular components, and signaling pathways were identified to assess the functional relevance of BFSP1 and its interacting proteins in LIHC.

### 2.10. siRNA Transfection Analysis

To suppress BFSP1 expression, HepG2 cells were transfected with either BFSP1-specific siRNA (filensin siRNA; Santa Cruz Biotechnology, Dallas, TX, USA, sc-62320) or a non-targeting control siRNA (sc-37007, Santa Cruz Biotechnology, Dallas, TX, USA) using Lipofectamine RNAiMAX transfection reagent (Invitrogen™, Thermo Fisher Scientific, Waltham, MA, USA), following the manufacturer’s protocol. Briefly, HepG2 cells were seeded into 6-well plates (Corning, Inc., Corning, NY, USA) at a density aimed to reach approximately 80% confluence on the day of transfection. siRNA duplexes were diluted to a final concentration of 50 nM in Opti-MEM Reduced Serum Medium (Gibco, Grand Island, NY, USA) and mixed using Lipofectamine RNAiMAX. After 10 min incubation at room temperature to allow complex formation, the siRNA-lipid complexes were gently added to the cells. The cells were incubated at 37 °C in a humidified atmosphere containing 5% CO_2_ for 24 or 48 h before harvesting for subsequent analyses. All experiments included non-targeting siRNA transfection controls and were conducted in triplicate.

### 2.11. Western Blotting Analysis

For protein expression analysis, cells were harvested and lysed using RIPA lysis buffer containing a protease inhibitor cocktail (Thermo Fisher Scientific). The lysates were centrifuged at 13,000× *g* for 15 min at 4 °C, and the supernatants were collected. Total protein concentration was measured using a bicinchoninic acid protein assay kit (Thermo Fisher Scientific), following the manufacturer’s protocol. Equal amounts of protein (20 μg per sample) were resolved by 8% sodium dodecyl sulfate-polyacrylamide gel electrophoresis and transferred onto polyvinylidene difluoride membranes (Millipore, Billerica, MA, USA). Membranes were blocked with 5% nonfat dry milk in Tris-buffered saline containing 0.1% Tween-20 for 1 h at room temperature, followed by overnight incubation at 4 °C with primary antibodies against BFSP1 (1:1000; Santa Cruz Biotechnology) and the loading control β-actin (1:5000; Thermo Fisher Scientific). After three washes with Tris-buffered saline containing 0.1% Tween-20, the membranes were incubated with horseradish peroxidase-conjugated secondary antibodies (1:5000; Cell Signaling Technology, Danvers, MA, USA) for 1 h at room temperature. Immunoreactive bands were detected using an enhanced chemiluminescence detection reagent (GE Healthcare, Chicago, IL, USA) according to the manufacturer’s protocol. Band intensities were quantified using ImageJ software (version 1.54p; National Institutes of Health, Bethesda, MD, USA) and normalized to β-actin.

### 2.12. Wound Healing Assay

The migratory capacity of HepG2 cells was evaluated in a scratch wound healing (scratch) assay. Following transfection with either control or BFSP1-specific siRNA, HepG2 cells were seeded into 6-well culture plates (Corning) and cultured until reaching approximately 90% confluency. A linear scratch was made at the center of each well using a sterile 200 µL pipette tip held perpendicular to the plate surface. Detached cells and debris were removed by gently rinsing the wells twice with phosphate-buffered saline (Gibco). The cells were incubated in serum-free medium to minimize wound closure attributable to cell proliferation. Phase-contrast images of the wound area were acquired at 0, 24, and 48 h post-scratch using an inverted phase-contrast microscope (Olympus, Tokyo, Japan) equipped with a digital camera. The wound width was measured at each time point, and the wound area was quantified using ImageJ software (version 1.54p). The percentage of wound closure was calculated using the following formula: wound closure (%) = ([area at 0 h × area at each subsequent time point]/area at 0 h) × 100.

### 2.13. Statistical Analysis

Comparative analyses of gene expression levels between tumor and normal tissues were conducted using integrated statistical tools available at TIMER2.0, UALCAN, and OncoDB. Survival analyses, including Kaplan–Meier curve generation and log-rank tests, were performed using the GSCA, GEPIA2, and MethSurv platforms. When appropriate, Cox proportional hazards regression models were applied to estimate the HR and 95% CIs, facilitating multivariate evaluation of prognostic factors. Correlation analyses, such as co-expression and immune infiltration assessments, were performed using either Pearson’s or Spearman’s correlation coefficients, depending on data distribution. To mitigate false-positives resulting from multiple hypothesis testing, Benjamini–Hochberg FDR correction was implemented, with statistical significance defined as *p* < 0.05 or FDR < 0.05. All analyses conformed to the methodological protocols of the respective web platforms, and the results were cross-validated when feasible to ensure reliability and robustness. The experimental data were statistically analyzed using GraphPad Prism software (version 10.4.1; GraphPad, Inc., San Diego, CA, USA). Data are expressed as the mean ± standard error of the mean from a minimum of three independent experiments. Differences between experimental groups were evaluated using two-way analysis of variance (ANOVA), followed by Tukey’s post hoc multiple comparison test to adjust for multiple testing. Statistical significance was set at *p* < 0.05. The number of replicates and specific statistical tests applied to each experiment are detailed in the corresponding figure legends.

## 3. Results

### 3.1. mRNA Expression of BFSP1 in LIHC

**Figure 1:**
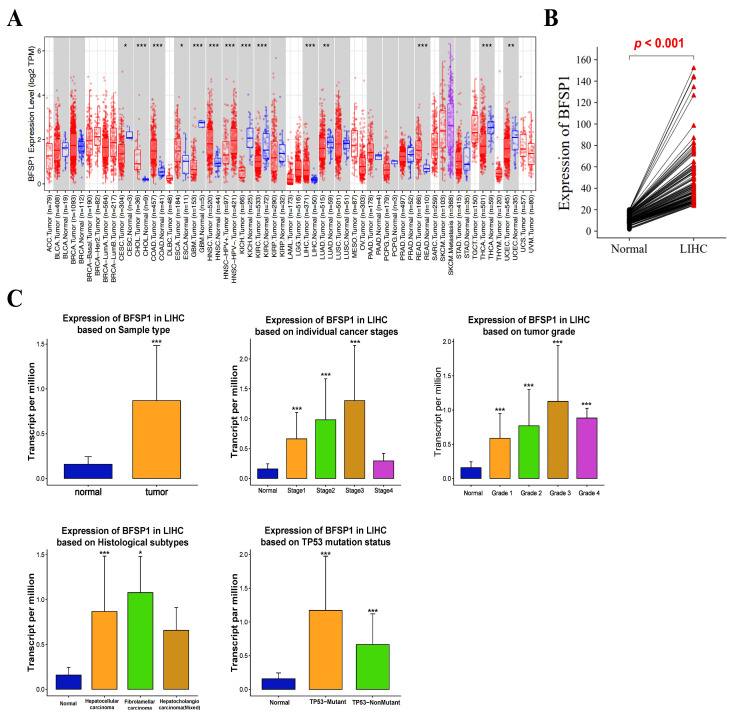
mRNA expression levels of BFSP1 in LIHC. (**A**) Comparison of BFSP1 expression between tumor and normal tissues in various cancer types. Blue indicates normal; red indicates tumor; purple indicates metastatic tumor. (**B**) Comparison of BFSP1 expression between LIHC and normal tissue. Each red triangle represents an individual tumor sample, and each dot represents an individual normal tissue sample. (**C**) Comparison of BFSP1 expression between clinicopathologic characteristics and normal conditions. * *p* < 0.05, ** *p* < 0.01, and *** *p* < 0.001.

To systematically characterize BFSP1 expression across diverse cancer types, pan-cancer analysis was performed using RNA sequencing data from TCGA accessed with the TIMER database. As shown in Figure 1A, BFSP1 mRNA expression was significantly elevated in several malignancies, including LIHC, cholangiocarcinoma, colon adenocarcinoma, esophageal carcinoma, head and neck squamous cell carcinoma, and rectal adenocarcinoma, compared with their corresponding normal tissues. In contrast, BFSP1 expression was significantly downregulated in other tumor types, such as cervical squamous cell carcinoma, endocervical adenocarcinoma, glioblastoma multiforme, HPV-negative head and neck squamous cell carcinoma, kidney chromophobe, renal clear cell carcinoma, lung adenocarcinoma, thyroid carcinoma, and uterine corpus endometrial carcinoma. To validate these findings in LIHC, BFSP1 expression was examined using the GSCA database, which independently confirmed significant upregulation of BFSP1 in LIHC tumor tissues relative to normal tissues (Figure 1B). Additionally, as shown in Figure 1C, BFSP1 expression in LIHC was assessed across various clinical subgroups using the UALCAN database. Elevated BFSP1 expression was significantly associated with several clinicopathological parameters, including primary tumor status, tumor stages I–III, tumor grades I–IV, histological subtype (particularly hepatocellular carcinoma), and *TP53* mutation status. Collectively, these results indicate that BFSP1 was markedly upregulated in LIHC across diverse clinicopathological contexts. The consistent overexpression observed across independent datasets supports the potential of BFSP1 as a pan-cancer biomarker and its diagnostic and prognostic relevance in LIHC.

### 3.2. Prognostic Value of BFSP1 Expression in LIHC

**Figure 2:**
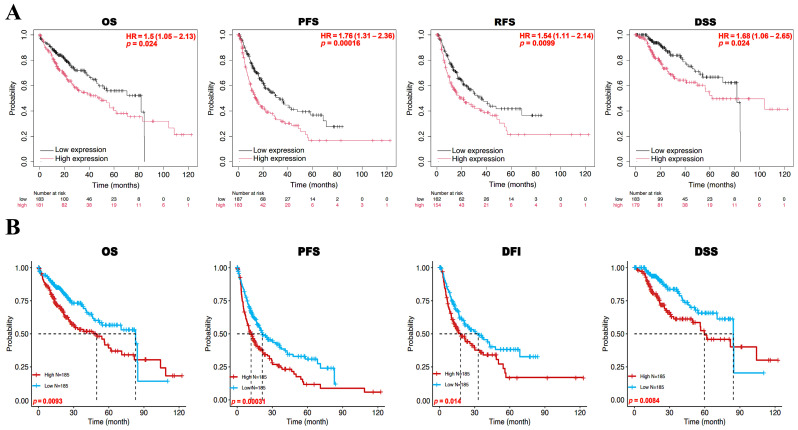
Prognostic significance of BFSP1 expression in LIHC. The prognostic value of BFSP1 expression was analyzed using Kaplan–Meier plotter (**A**) and GSCA (**B**). Overall survival, OS; progression-free survival, PFS; relapse-free survival, RFS; disease-free interval, DFI; disease-specific survival, DSS.

Survival analyses were conducted using Kaplan–Meier Plotter and the GSCA database to evaluate the prognostic significance of BFSP1 expression in patients in LIHC. Elevated BFSP1 expression was significantly correlated with unfavorable survival outcomes, including poorer OS (HR = 1.50, *p* = 0.024), progression-free survival (HR = 1.76, *p* = 0.00016), relapse-free survival (HR = 1.54, *p* = 0.0099), and disease-specific survival (DSS; HR = 1.68, *p* = 0.024) (Figure 2A). Further analysis revealed that high expression of BFSP1 correlated with poorer OS (*p* = 0.0093), progression-free survival (*p* = 0.00031), disease-free interval (*p* = 0.014), and DSS (*p* = 0.0084) (Figure 2B). We also investigated the correlation between BFSP1 expression and clinicopathological characteristics of LIHC. Higher BFSP1 expression was significantly associated with poorer survival outcomes across multiple clinicopathological subgroups. For OS, elevated BFSP1 expression was correlated with worse prognosis in patients with stage III disease (HR = 1.85, *p* = 0.039), stage III + IV disease (HR = 1.82, *p* = 0.037), non-vascular invasion (HR = 1.7, *p* = 0.047), Asian ethnicity (HR = 3.09, *p* = 0.00033), and hepatitis virus (yes, HR = 1.99, *p* = 0.039). In terms of relapse-free survival, a significantly poorer prognosis was observed in females (HR = 1.82, *p* = 0.048), stage I + II disease (HR = 1.52, *p* = 0.050), grade II disease (HR = 1.68, *p* = 0.036), AJCC_T I disease (HR = 1.74, *p* = 0.039), white ethnicity (HR = 1.65, *p* = 0.029), non-alcohol consumption (HR = 1.78, *p* = 0.011), and non-hepatitis virus (HR = 1.7, *p* = 0.036). For progression-free survival, significantly poorer prognosis were observed in both males (HR = 1.65, *p* = 0.00059) and females (HR = 1.96, *p* = 0.01), as well as in patents with stage I disease (HR = 1.7, *p* = 0.036), stage I + II disease (HR = 1.79, *p* = 0.0026), stage II + III disease (HR = 1.57, *p* = 0.027), stage III disease (HR = 1.82, *p* = 0.029), stage III + IV disease (HR = 1.81, *p* = 0.027), grade II disease (HR = 1.95, *p* = 0.0025), grade III disease (HR = 1.67, *p* = 0.048), AJCC_T I disease (HR = 1.84, *p* = 0.013), AJCC_T II disease (HR = 1.75, *p* = 0.05), non-vascular invasion (HR = 1.68, *p* = 0.022), white ethnicity (HR = 1.84, *p* = 0.0023), non-alcohol consumption (HR = 1.94, *p* = 0.0012), and non-hepatitis virus (HR = 1.96, *p* = 0.0022). For DSS, elevated BFSP1 expression was associated with poorer prognosis in males (HR = 1.92, *p* = 0.027), stage I + II disease (HR = 2.12, *p* = 0.04), stage II + III disease (HR = 2.09, *p* = 0.018), stage III disease (HR = 2.22, *p* = 0.027), stage III + IV disease (HR = 2.01, *p* = 0.044), grade II disease (HR = 2.27, *p* = 0.019), non-vascular invasion (HR = 2.27, *p* = 0.032), Asian (HR = 4.31, *p* = 0.00068), non-alcohol consumption (HR = 2.5, *p* = 0.0048), and hepatitis virus (yes, HR = 2.9, *p* = 0.014) (Table 1). Moreover, pan-cancer analysis demonstrated that increased BFSP1 expression was consistently associated with adverse survival outcomes across multiple cancer types (Table 2). To assess the prognostic impact of BFSP1 expression, survival analyses were conducted across eight independent datasets. High BFSP1 expression was significantly linked with poor OS in the GSE13507 (HR = 4.480, 95% CI: 1.720–11.660, *p* = 0.002), GSE12276 (HR = 1.260, 95% CI: 1.080–1.480, *p* = 0.004), GSE4475 (HR = 7.650, 95% CI: 1.380–42.490, *p* = 0.020), GSE1379 (HR = 1.830, 95% CI: 1.080–3.100, *p* = 0.025), GSE1378 (HR = 1.510, 95% CI: 1.040–2.180, *p* = 0.031), and GSE8970 (HR = 1.800, 95% CI: 1.010–3.220, *p* = 0.046) cohorts. In contrast, an inverse correlation with elevated BFSP1 expression was observed in the GSE31210 (HR = 0.760, 95% CI: 0.600–0.960, *p* = 0.020) and E-TABM-158 (HR = 0.110, 95% CI: 0.010–1.000, *p* = 0.049) datasets, suggesting a potential protective role in these populations (Table 2). Collectively, these findings indicate that elevated BFSP1 expression serves as a robust marker of poor prognosis in LIHC, emphasizing its potential as a significant prognostic biomarker.

### 3.3. DNA Methylation and Prognosis of BFSP1 in LIHC

**Figure 3:**
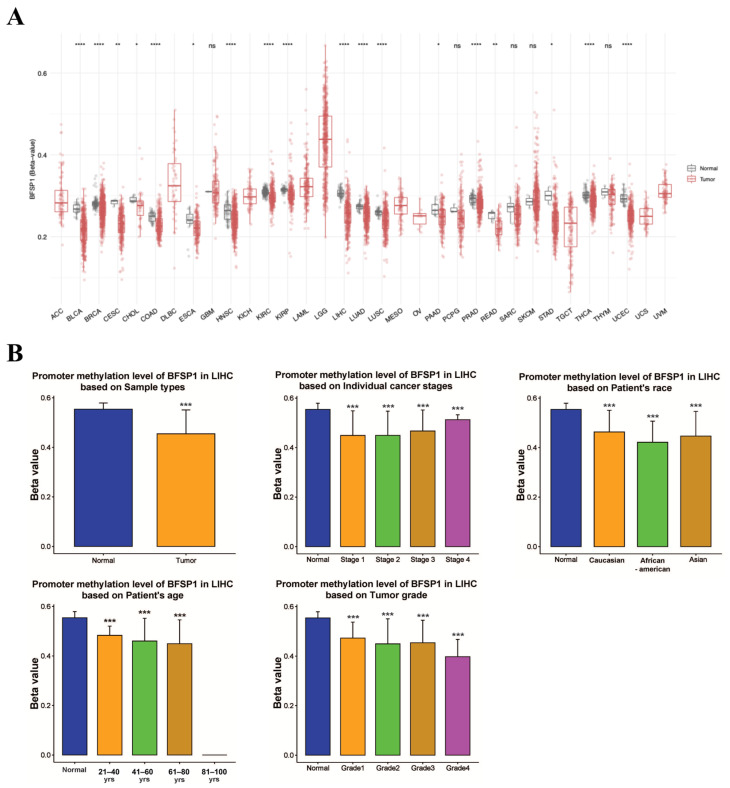
DNA methylation levels of BFSP1 in LIHC. (**A**) Comparison of DNA methylation of BFSP1 expression between tumor and normal tissues across various cancer types. “ns” indicates “not significant”. (**B**) Comparison of BFSP1 DNA methylation between LIHC and normal tissues across clinicopathologic characteristics. * *p* < 0.05, ** *p* < 0.01, *** *p* < 0.001. and **** *p* < 0.0001.

**Figure 4:**
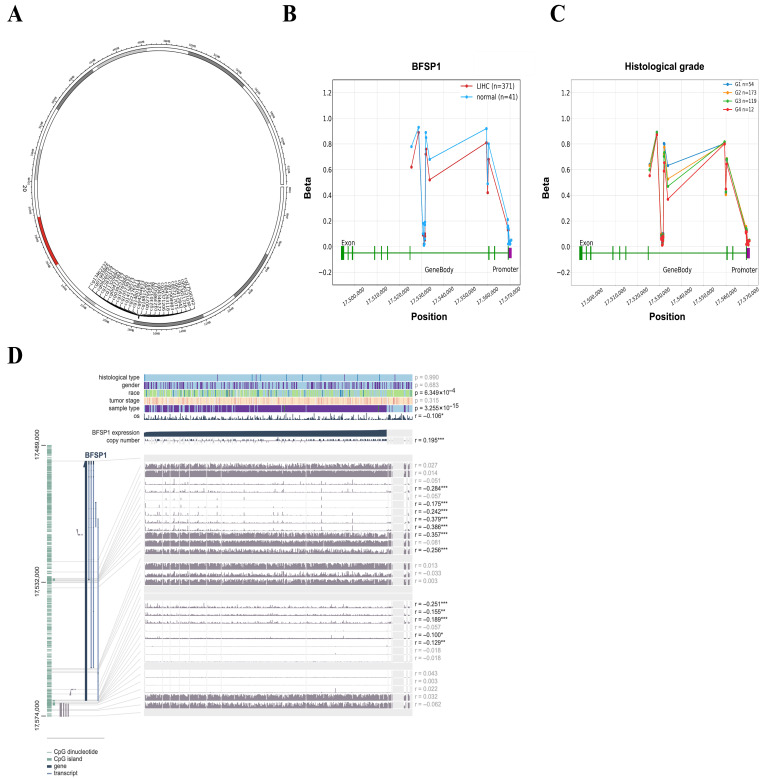
DNA methylation and correlation of BFSP1 in LIHC. (**A**) DNA methylation probes mapped to BFSP1 in LIHC. Grey segments indicate chromosomal ideograms, and colored regions showed methylation-associated loci. (**B**) DNA methylation of BFSP1 between LIHC and normal tissues. The downward green vertical lines indicate exon boundaries and the purple vertical bar denotes the promoter region near the transcription start site. (**C**) Comparison between DNA methylation of BFSP1 and histological grade in LIHC. The downward green vertical lines indicate exon boundaries and the purple vertical bar denotes the promoter region near the transcription start site. (**D**) Correlation of BFSP1-associated probes in LIHC. * *p* < 0.05, ** *p* < 0.01, and *** *p* < 0.001.

**Figure 5:**
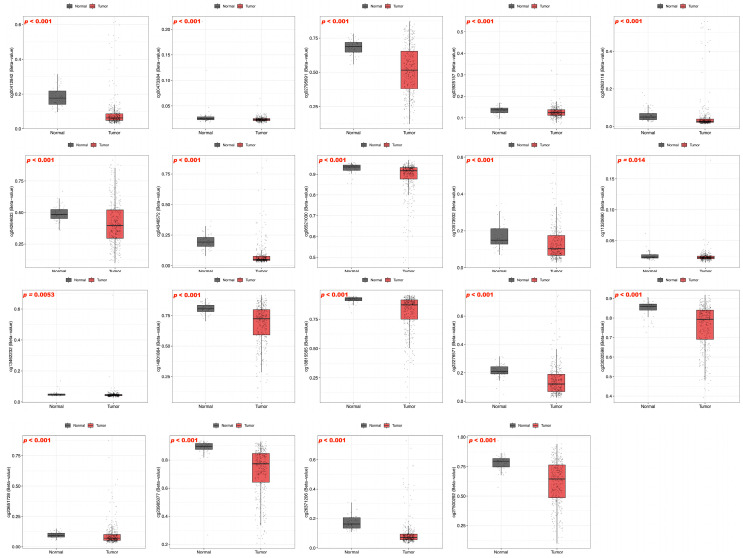
Comparison of BFSP1-associated DNA methylation probes between LIHC and normal tissues. Dots indicate the β-methylation value of each individual sample.

**Figure 6:**
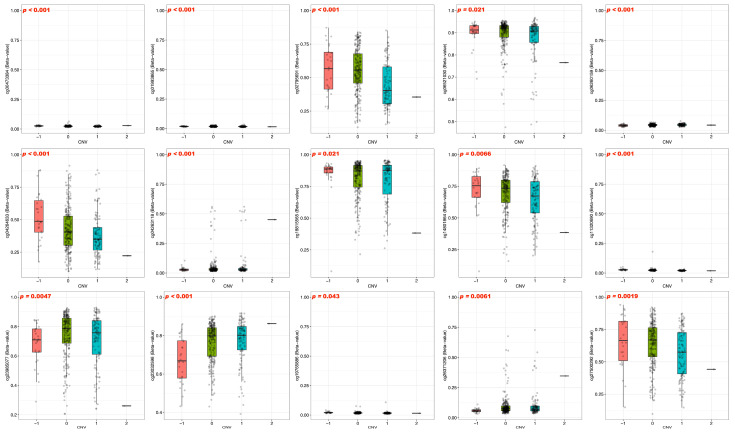
CNVs of BFSP1-associated DNA methylation probes in LIHC. Circles represent the β-methylation value of each individual sample.

**Figure 7:**
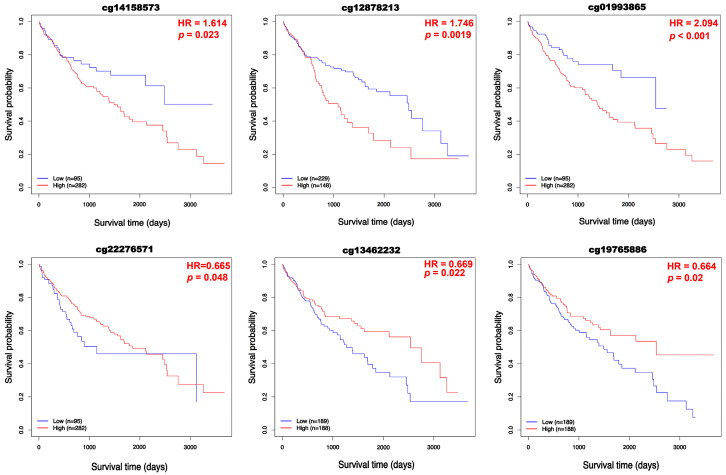
Prognostic significance of BFSP1-associatied DNA methylation probes in LIHC.

To examine the epigenetic regulation of BFSP1 in LIHC, comprehensive DNA methylation analyses were conducted using multiple public databases including UALCAN, OncoDB, SMART, and MEXPRESS. The results revealed significant hypomethylation of the BFSP1 promoter region in several cancer types, including LIHC (Figure 3A). Furthermore, BFSP1 methylation and expression levels were significantly associated with various clinicopathological features such as primary tumor status, pathological stage (I–IV), histological grade (I–IV), and *TP53* mutation status (Figure 3B). Among the 26 BFSP1-associated probe sites, 13 demonstrated significant differences in methylation between LIHC and normal tissues. Notably, hypomethylation was observed at several probes, including cg27500292 (*p* < 0.001), cg06521030 (*p* < 0.001), cg00412842 (*p* < 0.001), cg26371206 (*p* < 0.001), cg04346572 (*p* < 0.001), cg23985077 (*p* < 0.001), cg23032598 (*p* < 0.001), cg02795691 (*p* < 0.001), cg18815565 (*p* < 0.001), cg04264633 (*p* < 0.001), cg14801864 (*p* < 0.001), and cg22276571 (*p* < 0.001), and cg10573932 (*p* = 0.008) (Figure 4A,B and Table 3 and Table 4). Furthermore, methylation profiling of BFSP1 according to histological grade in LIHC revealed distinct epigenetic patterns across the promoter and gene body regions (Figure 4C). Correlation analysis demonstrated that BFSP1 expression was significantly and inversely correlated with DNA methylation at multiple CpG sites. Notably, numerous CpG sites in both the promoter and gene body regions exhibited negative correlations, including cg14801864 (R = −0.379, *p* < 0.001), cg04346572 (R = −0.386, *p* < 0.001), cg23985077 (R = −0.357, *p* < 0.001), cg00412842 (R = −0.284, *p* < 0.001), cg04263118 (R = −0.242, *p* < 0.001), cg02795691 (R = −0.256, *p* < 0.001), cg22276571 (R = −0.251, *p* < 0.001), cg07904983 (R = −0.175, *p* < 0.001), cg14158573 (R = −0.155, *p* < 0.01), cg13462232 (R = −0.189, *p* < 0.01), cg03925157 (R = −0.100, *p* < 0.05), and cg00473384 (R = −0.129, *p* < 0.05) (Figure 4D). Additionally, multiple probes within the BFSP1 promoter and gene body regions exhibited significant hypomethylation in LIHC tissues compared with normal tissues, including cg00412842 (*p* < 0.001), cg00473384 (*p* < 0.001), cg02795691 (*p* < 0.001), cg03925157 (*p* < 0.001), cg04264633 (*p* < 0.001), cg04346572 (*p* < 0.001), cg06521030 (*p* < 0.001), cg13462232 (*p* < 0.001), cg11320690 (*p* = 0.014), cg14801864 (*p* < 0.001), cg18815565 (*p* < 0.001), cg22276571 (*p* < 0.001), cg23032598 (*p* < 0.001), cg23651728 (*p* < 0.001), cg23985077 (*p* < 0.001), cg26371206 (*p* < 0.001), and cg27500292 (*p* < 0.001) (Figure 5). To investigate the relationship between BFSP1 CNVs and site-specific DNA methylation patterns in LIHC, we conducted integrative analysis combining CNV and methylation data across multiple CpG sites within the BFSP1 genomic locus. The analysis revealed a consistent and significant association between CNV status and methylation levels at numerous loci, suggesting interplay between genomic dosage and epigenetic regulation. Notably, several CpG probes exhibited significant methylation alterations in association with CNV states, including cg00412842 (*p* < 0.001), cg019933865 (*p* < 0.001), cg02795691 (*p* < 0.001), cg06521030 (*p* = 0.021), cg06260159 (*p* < 0.001), cg04264633 (*p* < 0.001), cg04263118 (*p* < 0.001), cg18815565 (*p* = 0.021), cg14801864 (*p* = 0.0066), cg11320690 (*p* < 0.001), cg23985077 (*p* = 0.0047), cg23032598 (*p* < 0.001), cg19765886 (*p* = 0.043), cg26371206 (*p* = 0.0061), and cg27500292 (*p* = 0.0019) (Figure 6). To evaluate the prognostic significance of BFSP1 methylation-associated probes in LIHC, survival analysis was conducted using the MethSurv database. Hypermethylated probes, including cg14158573 (HR = 1.614, *p* = 0.023), cg12878213 (HR = 1.746, *p* = 0.0019), and cg18815565 (HR = 2.094, *p* < 0.001) were significantly associated with poor prognosis in LIHC. In addition, hypomethylated probes, such as cg22276571 (HR = 0.665, *p* = 0.048), cg13462232 (HR = 0.669, *p* = 0.022), and cg19765886 (HR = 0.664, *p* = 0.02), were also associated with poor prognosis in LIHC (Figure 7). Collectively, these findings suggest that DNA methylation profiling of BFSP1 may serve as a potential epigenetic biomarker and could contribute to risk stratification, molecular subtyping, and developing precise epigenetic therapies for LIHC, although further validation is required.

### 3.4. Correlation Between BFSP1 and Immune Cell Infiltration in LIHC

**Figure 8:**
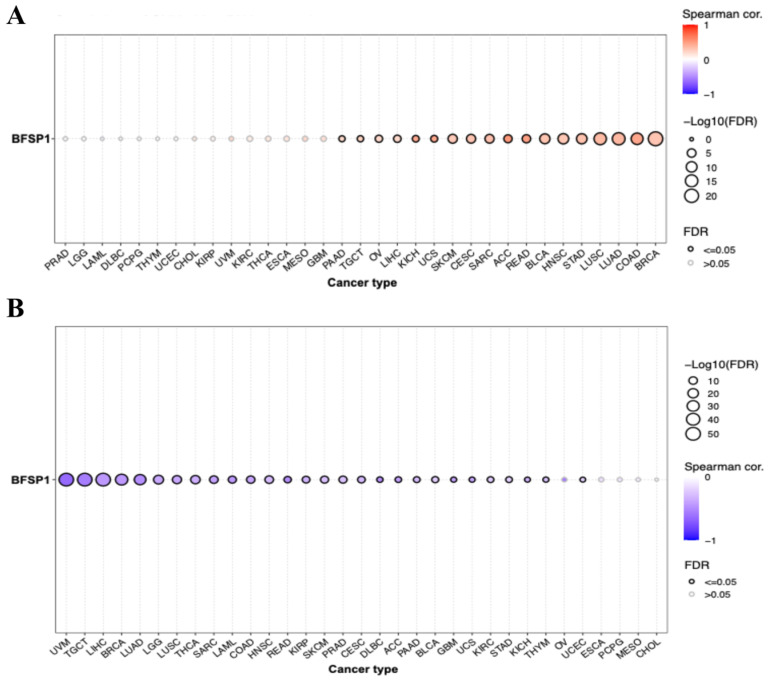
Correlation of BFSP1 CNV (**A**) and DNA methylation (**B**) in multiple cancer types, including LIHC.

To further evaluate the regulatory mechanisms underlying BFSP1 expression across cancer types, we systematically examined the correlation between CNVs, DNA methylation, and BFSP1 mRNA levels. CNV analysis revealed a positive correlation between BFSP1 mRNA expression and multiple cancers, including LIHC (Figure 8A). Conversely, DNA methylation demonstrated a strong negative correlation with BFSP1 mRNA expression in several cancer types, including LIHC (Figure 8B).

**Figure 9:**
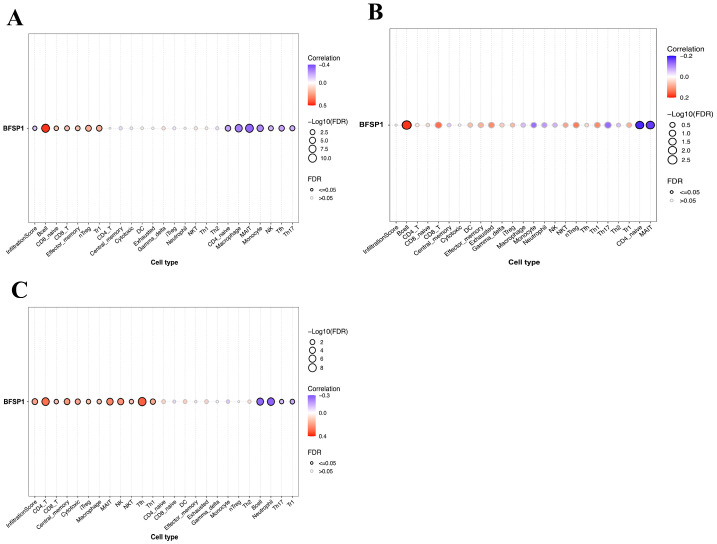
Correlation between BFSP1 expression and TIICs using the GSCA database in LIHC. (**A**) Expression of BFSP1, (**B**) CNV of BFSP1, (**C**) DNA methylation of BFSP1.

**Figure 10:**
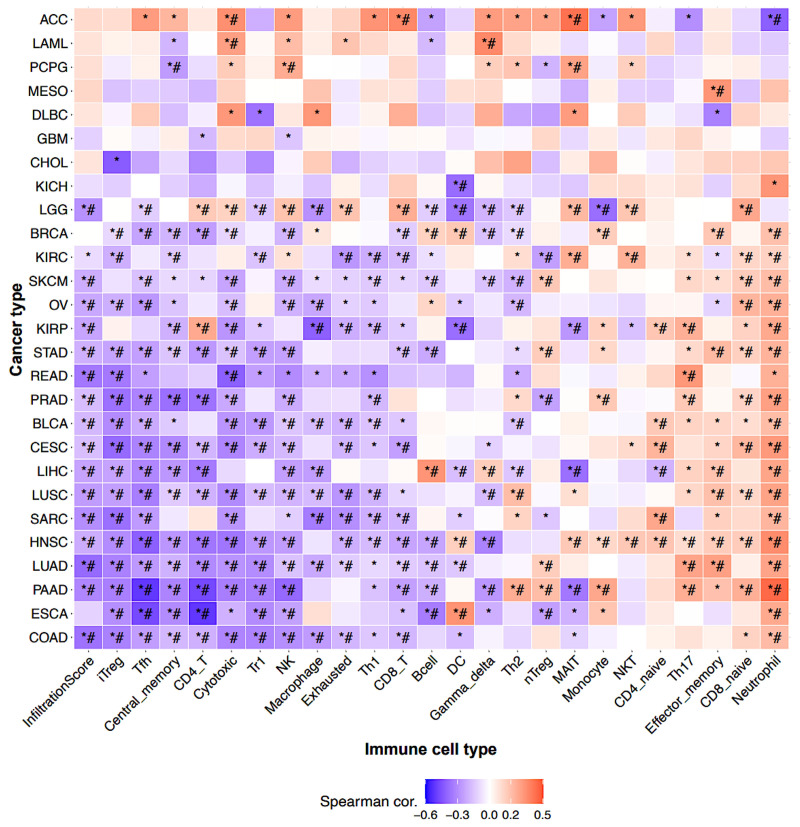
Correlation between BFSP1 expression and TIICs in various cancer types including LIHC determined using the GSCA database. * *p* ≤ 0.05, # FDR ≤ 0.05.

Correlation analysis between BFSP1 and TIICs in LIHC revealed distinct patterns depending on the analysis type (Figure 9 and Table 5). Evaluation of gene expression showed that BFSP1 was positively correlated with B cells (R = 0.46, *p* < 0.001), regulatory T cells (Tregs) (R = 0.21, *p* < 0.001), type 1 Treg cells (R = 0.21, *p* < 0.001), CD8+ T cells (R = 0.16, *p* < 0.001), CD8-naïve T cells (R = 0.16, *p* < 0.001), and natural killer (NK) T cells (R = 0.15, *p* < 0.001). In contrast, negative correlations were observed with mucosal-associated invariant T (MAIT) cells (R = −0.33, *p* < 0.001), macrophages (R = −0.29, *p* < 0.001), monocytes (R = −0.25, *p* < 0.001), T follicular helper cells (R = –0.20, *p* < 0.001), CD4-naïve T cells (R = −0.19, *p* < 0.001), Th17 (R = −0.17, *p* < 0.001), and NK cells (R = −0.16, *p* < 0.001). Analysis of BFSP1 CNVs in relation to immune cell infiltration in LIHC revealed positive correlation with B cells (R = 0.18, *p* < 0.001), Tregs (R = 0.13, *p* = 0.01), CD8+ T cells (R = 0.14, *p* = 0.006), and Th1 cells (R = 0.12, *p* = 0.02). In contrast, negative correlations were observed with CD4-naïve T cells (R = −0.18, *p* < 0.001), MAIT cells (R = −0.17, *p* < 0.001), Th17 cells (R = −0.13, *p* = 0.02), and monocytes (R = −0.13, *p* = 0.02). Similarly, BFSP1 methylation was significantly associated with immune cell infiltration in LIHC. BFSP1 methylation levels were positively correlated with T follicular helper cells (R = 0.31, *p* < 0.001), CD4+ T cells (R = 0.28, *p* < 0.001), MAIT cells (R = 0.25, *p* < 0.001), NK cells (R = 0.22, *p* < 0.001), Th1 cells (R = 0.18, *p* < 0.001), inducible Tregs (R = 0.14, *p* = 0.008), macrophages (R = 0.13, *p* = 0.01), CD8+ T cells (R = 0.13, *p* = 0.01), and NKT cells (R = 0.13, *p* = 0.01). In contrast, negative correlations were observed with neutrophils (R = −0.27, *p* < 0.001), B cells (R = −0.27, *p* < 0.001), Tr1 cells (R = −0.15, *p* = 0.003), and Th17 cells (R = −0.11, *p* = 0.03). Further analysis demonstrated that, in LIHC, BFSP1 expression exhibited a strong positive correlation with B cells, gamma delta T cells, effector memory T cells, and neutrophils. In contrast, negative correlations were observed between BFSP1 expression and infiltration of inducible Tregs, T follicular helper cells, central memory T cells, CD4+ T cells, NK cells, macrophages, MAIT cells, CD4-naïve T cells, dendritic cells, and Th2 cells (Figure 10). Similar patterns of positive and negative correlations between BFSP1 expression and immune cell infiltration have been observed across multiple other cancer types. Collectively, these findings suggest that both genomic and epigenetic alterations in BFSP1 shape the immune landscape of LIHC. Specifically, genomic changes in BFSP1 (such as CNVs) may influence the overall composition of the tumor immune microenvironment, thereby affecting immune surveillance and tumor progression. Similarly, epigenetic regulation through DNA methylation may differentially modulate the infiltration of specific immune cell subsets, ultimately affecting immune responses and disease outcomes in LIHC.

### 3.5. Association Between BFSP1 Expression and Drug Sensitivity in LIHC

**Figure 11:**
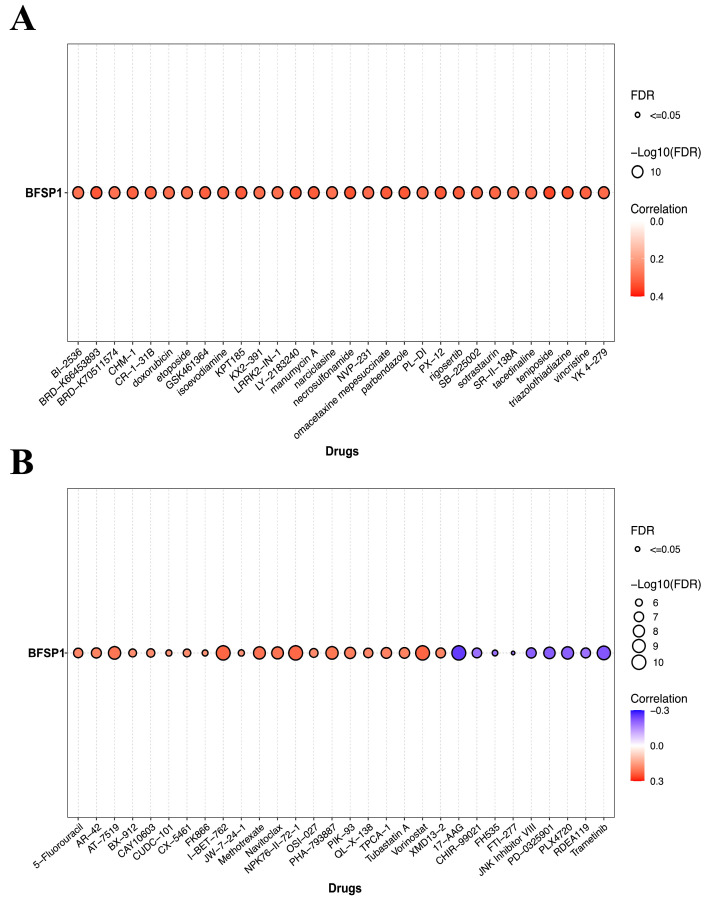
Correlation between BFSP1 expression and drug sensitivity. (**A**) Correlation between BFSP1 and drug sensitivity using GDSC in the GSCA database (**B**) Correlation between BFSP1 and drug sensitivity using CTRP in the GSCA database.

To examine the relationship between BFSP1 expression and drug response in LIHC, drug sensitivity analyses were performed using data from the GDSC and CTRP databases. Analysis of the GDSC dataset revealed positive correlations between BFSP1 expression and enhanced sensitivity to multiple anticancer agents, including BI-2536, BRD-K66433693, BRD-K70511574, CHM-1, CR-1-31B, doxorubicin, etoposide, GSK461364, isoevodiamine, KPT185, KX2-391, LRRK2-IN-1, LY-2183240, manumycin A, narciclasine, necrosulfonamide, NVP-231, omacetaxine mepesuccinate, parbendazole, PL-DI, PX-12, rigosertib, SB-225002, sotrastaurin, SRI-31138A, tacedinaline, teniposide, triazolothiadiazine, vincristine, and YK-4-279 (Figure 11A). In contrast, drug sensitivity analysis based on the CTRP dataset revealed both positive and negative correlations between BFSP1 expression and response to various anticancer agents. Positive correlations were identified for 5-fluorouracil, AR-42, AT-7519, BX-912, CAY10603, CUDC-101, CX-5461, FK866, I-BET-762, JW-7-24-1, methotrexate, navitoclax, NPK76-II-72-1, OSI-027, PHA-793887, QL-X-138, TPCA-1, tubastatin A, vorinostat, and XMD13-2. In contrast, negative correlations were observed for 17-AAG, CHIR-99021, FTl535, JNK inhibitor VIII, PD-0325901, PLX4720, RDEA119, and trametinib, indicating resistance associated with elevated BFSP1 expression (Figure 11B). Collectively, these findings suggest that BFSP1 expression may be used as a predictive biomarker for drug response in LIHC, thereby offering valuable insights into the development of personalized therapeutic strategies.

### 3.6. Interactions Between BFSP1 Expression and Chemicals in LIHC

The CTD was used to further evaluate the regulatory effects of chemical compounds on BFSP1 expression. We identified 44 chemicals as modulators of BFSP1 expression, of which 26 were associated with upregulation and 18 with downregulation (Table 6). Notably, compounds correlated with increased BFSP1 expression encompassed a diverse range of environmental and pharmacological agents, such as 2,3′,4,4′,5-pentachlorobiphenyl, acetamide, afuresertib, aristolochic acid, benzo(b)fluoranthene, copper sulfate, folic acid, gentamicins, hexachlorocyclohexane, lipopolysaccharides, methamphetamine, particulate matter, propylthiouracil, sodium arsenite, tetrachlorodibenzodioxin, thioacetamide, trichloroethylene, triptolide, and vinclozolin. In contrast, compounds associated with decreased BFSP1 expression included acetaminophen, acrylamide, belinostat, bilirubin, bisphenol A, dichlorodiphenyl dichloroethylene, dietary fats, domoic acid, ICG 001, sunitinib, testosterone, thalidomide, tobacco smoke pollution, tretinoin, zinc sulfate, and zoledronic acid. To further characterize the chemical interaction profile of BFSP1, a similarity index analysis was performed to identify genes with common interacting chemicals with BFSP1 based on CTD (Table 7). Several genes, including *CCDC88B*, *CCDC102A*, *OLFM2*, *SYTL3*, *CPNE5*, *LYL1*, *MGAM*, *PRX*, *SHANK1*, *KCTD13*, *PLEKHA4*, *ANKS6*, *SH3RF2*, *KCNAB1*, *KCNH4*, *CASS4*, *C5AR2*, *MPDU1*, *ARHGAP27*, and *NPTXR*, exhibited high similarity index with BFSP1, indicating substantial overlap in their chemical interaction profiles. Among these, *MGAM* and *KCNAB1* shared the largest number of chemicals with BFSP1 (n = 30), whereas *CCDC88B*, *CPNE5*, *SHANK1*, and *SH3RF2* shared 27 chemicals. These findings suggest that BFSP1 and its co-expressed or functionally related genes are targeted or regulated by similar environmental or pharmacological agents, underscoring their potential significance in toxicogenomic pathways and cancer susceptibility.

### 3.7. Co-Expression and Functional Enrichment Analysis of BFSP1 in LIHC

**Figure 12:**
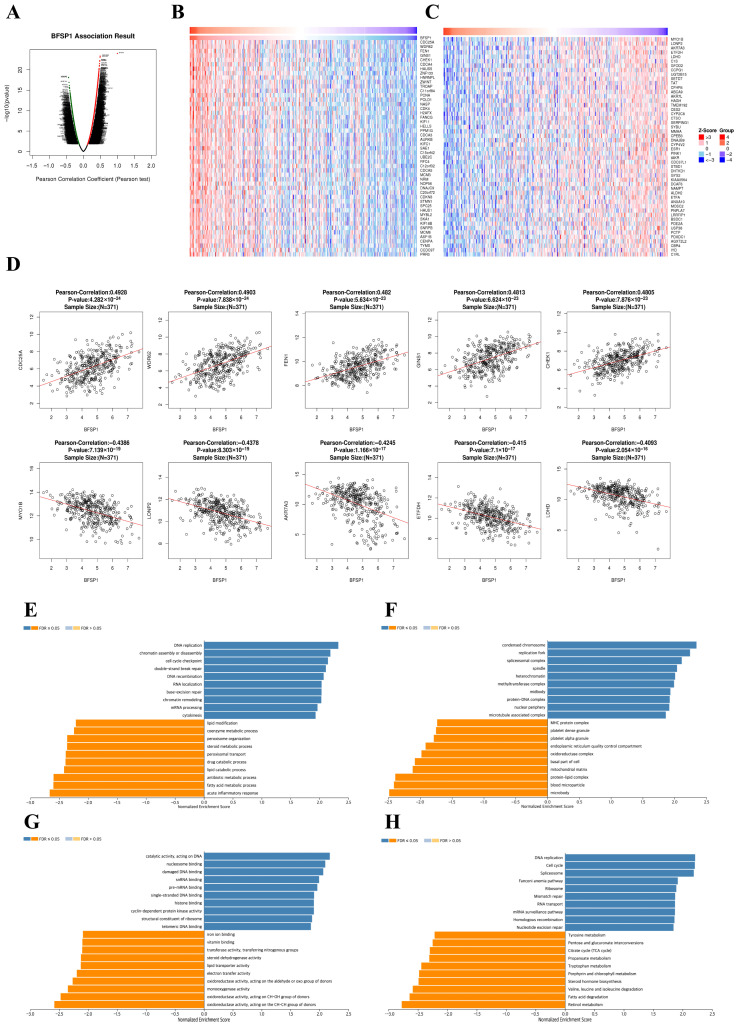
Co-expression and functional enrichment for BFSP1 expression in LIHC. (**A**) Genes positively and negatively correlated with BFSP1 expression in LIHC. Dark red dots: positive correlation, dark green: negative correlation. (**B**) Heat map showing the top 50 genes positively correlated with BFSP1 expression in LIHC. (**C**) Heat map showing the top 50 genes negatively correlated with BFSP1 expression in LIHC. (**D**) Correlation of the top five genes positively and negatively correlated with BFSP1 expression in LIHC. Line: regression line. (**E**) GO-biological process related to the co-expressed genes of BFSP1 determined using GSEA. (**F**) Enriched GO-cellular component related to the co-expressed genes of BFSP1 by GSEA. (**G**) Enriched GO-molecular function related to the co-expressed genes of BFSP1 by GSEA. (**H**) Enriched GO-KEGG pathway related to the co-expressed genes of BFSP1 by GSEA. Dark blue and orange indicate an FDR ≤ 0.05.

Comprehensive co-expression analysis was conducted using the LinkedOmics database to explore the biological functions of BFSP1 in LIHC. This analysis identified 11,994 genes positively correlated with BFSP1 expression (represented by dark red dots in Figure 12A) and 7928 genes negatively correlated (represented by dark green dots). These results indicate that BFSP1 is intricately associated with an extensive gene network and modulates diverse biological processes in LIHC. Heatmap visualization was used to highlight the top 50 genes with the most significant positive and negative correlations with BFSP1 (Figure 12B,C). The strongest positive correlations were detected with *CDC25A* (R = 0.4928, *p* < 0.001), *WDR62* (R = 0.4903, *p* < 0.001), *FEN1* (R = 0.482, *p* < 0.001), *GINS1* (R = 0.483, *p* < 0.001), and *CHEK1* (R = 0.4805, *p* < 0.001). In contrast, strong negative correlations were observed with *MYO1B* (R = −0.4386, *p* < 0.001), *LONP2* (R = −0.4378, *p* < 0.001), *AKR7A3* (R = −0.4245, *p* < 0.001), *ETFDH* (R = −0.15, *p* < 0.001), and *LDHD* (R = −0.4093, *p* < 0.001) (Figure 12D). In addition, GSEA was performed using the LinkedOmics platform to further elucidate the functional significance of BFSP1 and its co-expressed genes in LIHC. The analysis revealed significant enrichment of BFSP1-correlated genes in several biological processes, cellular components, molecular functions, and KEGG pathways. Within the biological process category, BFSP1 co-expressed genes were predominantly enriched in pathways associated with chromosome segregation, mitotic nuclear division, DNA replication, nuclear chromosome segregation, and mitotic sister chromatid segregation, indicating their pivotal role in cell cycle regulation (Figure 12E). Regarding cellular components, the most enriched terms encompassed chromosomal regions, spindles, condensed chromosomes, kinetochores, and nuclear chromosomes, further corroborating their involvement in the mitotic machinery (Figure 12F). Significantly enriched terms in the molecular function category included microtubule binding, catalytic activity against DNA, single-stranded DNA binding, tubulin binding, and DNA helicase activity (Figure 12G). KEGG pathway analysis revealed significant enrichment in pathways related to the cell cycle, DNA replication, homologous recombination, Fanconi anemia pathway, and mismatch repair (Figure 12H). Collectively, these findings suggest that BFSP1 and its co-expressed genes are closely associated with the regulation of cell cycle progression, chromosomal stability, and DNA repair mechanisms in LIHC.

### 3.8. Prognostic Value of BFSP1-Associated Genes in LIHC

**Figure 13:**
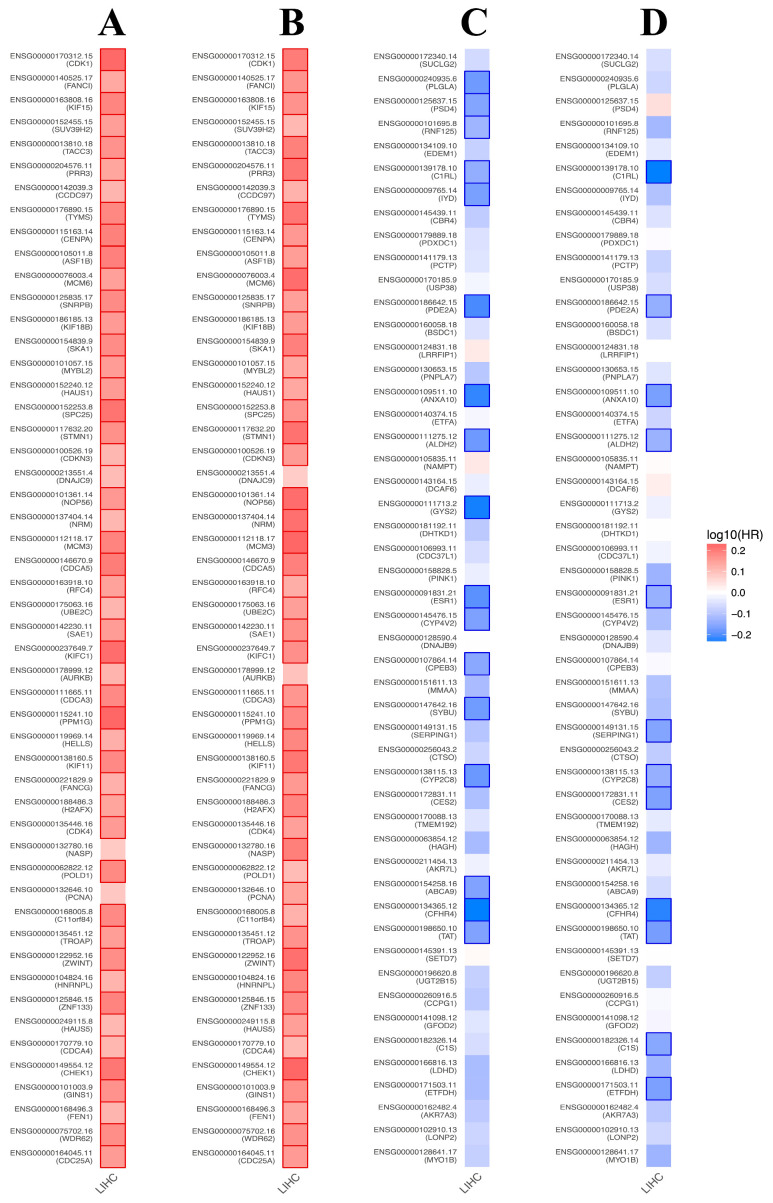
Prognostic significance of BFSP1-related genes in LIHC. Survival map of the positively correlated genes of BFSP1 for OS (**A**) and DFS (**B**). Survival map of the negatively correlated genes with BFSP1 expression for OS (**C**) and DFS (**D**). Heat map showing the log^10^ (HR) of genes in LIHC. Squares with a bold border represent *p* < 0.05 in survival analysis.

**Figure 14:**
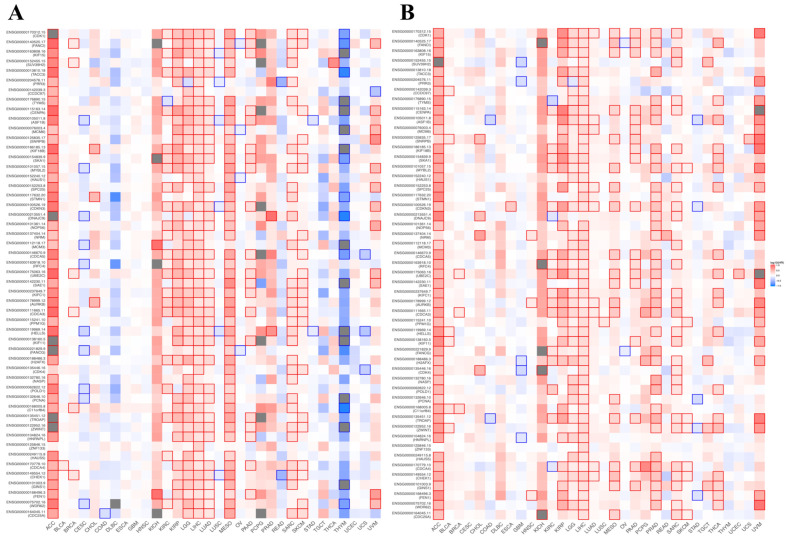

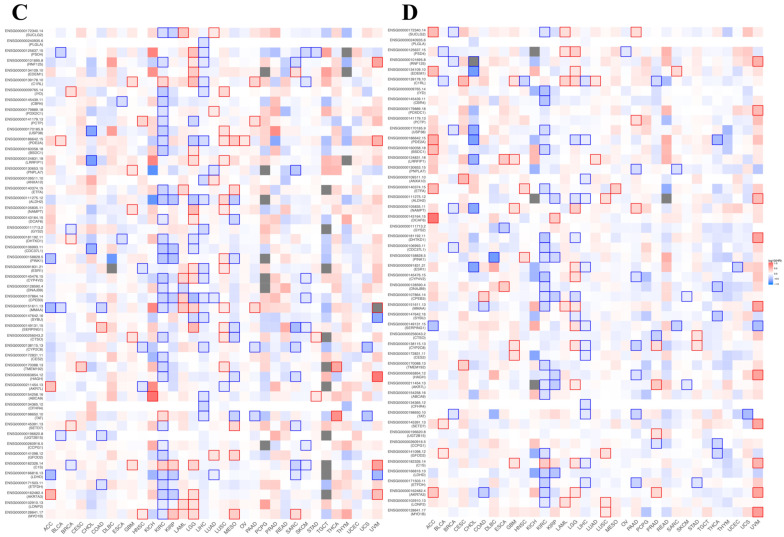
Prognostic significance of BFSP1-related genes in various cancer types. Survival map of the positively correlated genes of BFSP1 for OS (**A**) and DFS (**B**). Survival map of the negatively correlated genes with BFSP1 expression for OS (**C**) and DFS (**D**). Heat map showing the log^10^ (HR) of genes in various cancer types. Squares with a bold border represent *p* < 0.05 in survival analysis.

To assess the prognostic significance of BFSP1-associated genes in LIHC, we analyzed data from the GEPIA2 database. The findings indicated that genes positively correlated with BFSP1 act as high-risk factors for LIHC. Specifically, among these positively correlated genes, 48 were associated with a high HR for OS (Figure 13A), whereas 42 genes exhibited a high HR for DFS (Figure 13B). Conversely, genes negatively correlated with BFSP1 exhibited a more favorable prognostic profile, with 17 genes linked to a low HR for OS (Figure 13C) and 17 genes associated with a low HR for DFS (Figure 13D). To visualize the prognostic relevance of BFSP1-associated genes across various cancer types, including LIHC, heatmap analyses were performed for both high- and low-HR gene sets (Figure 14A–D). Collectively, these findings suggest that BFSP1 and its positively correlated gene set are significantly associated with poor prognosis in LIHC, supporting their potential as prognostic biomarkers and therapeutic targets.

### 3.9. Gene–Gene Interaction Network Analysis of BFSP1

**Figure 15:**
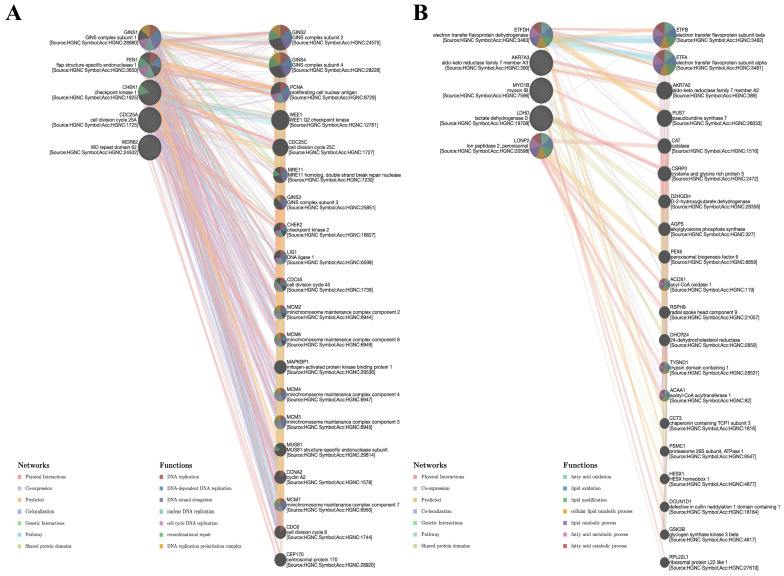
Gene–gene interaction network analysis of BFSP1. (**A**) Genes positively correlated with BFSP. (**B**) Genes negatively correlated with BFSP1.

To comprehensively characterize the gene–gene interaction network of BFSP1, we performed network analysis using the GeneMANIA platform. This approach yielded an extensive network, revealing significant associations between BFSP1 and various functionally relevant genes, encompassing both direct physical interactions and indirect relationships mediated through co-expression, genetic interactions, and shared protein domains. To identify the biological pathways underlying the BFSP1 co-expression network in LIHC, we performed functional enrichment analyses of genes exhibiting strong positive or negative correlations with BFSP1. Genes positively correlated with BFSP1 were significantly related to DNA replication, mitotic cell cycle progression, DNA strand elongation, multiple DNA repair pathways, as well as the DNA replication pre-initiation complex. These genes primarily participate in the maintenance of genomic stability and cell proliferation, as reflected by the dense physical interactions and co-expression modules identified within the network (Figure 15A). In contrast, genes negatively correlated with BFSP1 were primarily associated with fatty acid oxidation, lipid metabolic processes, peroxisomal and mitochondrial metabolism, and the regulation of redox homeostasis. Notably, key annotated functions included fatty acid catabolic and lipid metabolic processes, indicating that reduced BFSP1 expression is correlated with enhanced metabolic and oxidative regulatory activities (Figure 15B). Collectively, these analyses suggest that BFSP1 upregulation is functionally linked to cell cycle progression and DNA replication/repair mechanisms, whereas its downregulation corresponds to increased cellular metabolism and fatty acid oxidation. These distinct co-expression and interaction networks provide mechanistic insights into the multifaceted roles of BFSP1 in LIHC tumor biology.

### 3.10. Protein–Protein Interaction and Functional Enrichment Analysis of BFSP1 in LIHC

**Figure 16:**
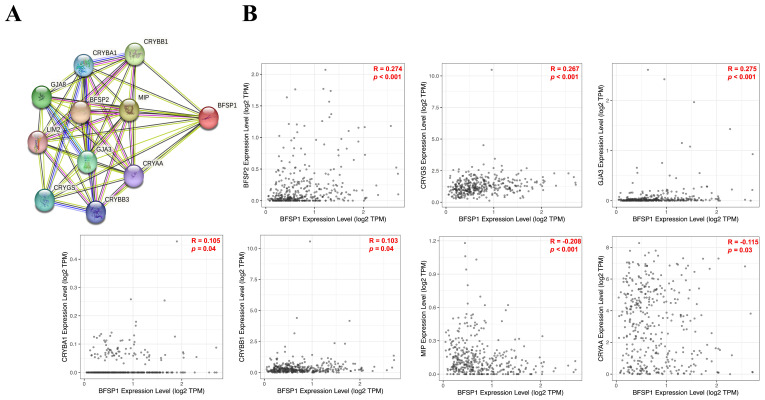

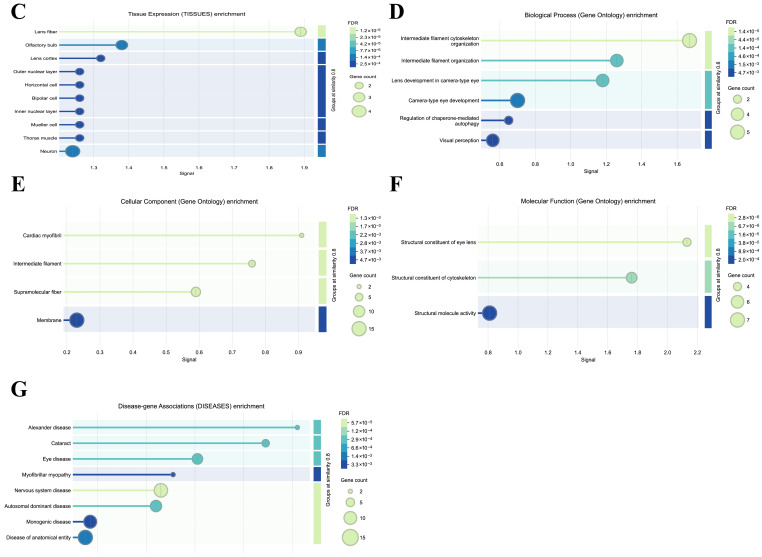
PPI network and functional enrichment analysis of BFSP1. (**A**) PPIs of BFSP1. Edge colors indicate different types of interaction evidence—red: fusion, green: neighborhood, blue: co-occurrence, purple: experimental, yellow: text-mining, light blue: database, black: co-expression. (**B**) Correlation of the BFSP1-associated PPI network. Dots represent the expression level in each individual sample. (**C**) Tissue expression related to co-expressed proteins of BFSP1. (**D**) Gene Ontology (GO) biological processes of the BFSP1-associated PPI network. (**E**) GO cellular components of the BFSP1-associated PPI network. (**F**) GO molecular functions of the BFSP1-associated PPI network. (**G**) Disease-related genes in the BFSP1-associated PPI network.

To elucidate the PPI network of BFSP1 in LIHC, we constructed a comprehensive PPI network using the STRING database (Figure 16A). This analysis identified several direct and indirect interactors of BFSP1, including BFSP2, CRYGS, GJA3, CRYAA, CRYBA1, CRYBB1, CRYBB3, LIM2, MIP, and GJA8, many of which are functionally associated with cytoskeletal organization, cellular junctions, and lens development. Correlation analyses revealed significant positive associations between BFSP1 expression and several interacting partners, including BFSP2 (R = 0.274, *p* < 0.001), CRYGS (R = 0.267, *p* < 0.001), GJA3 (R = 0.275, *p* < 0.001), CRYBA1 (R = 0.105, *p* = 0.04), and CRYBB1 (R = 0.103, *p* = 0.04), while negative correlations were observed with MIP (R = −0.208, *p* < 0.001) and CRYAA (R = −0.115, *p* = 0.03) (Figure 16B). GO and pathway enrichment analyses were performed to further characterize the biological functions associated with the BFSP1 PPI network in LIHC. Tissue expression analysis revealed that BFSP1 and its interacting partners were predominantly expressed in lens fiber cells, highlighting their critical roles in ocular tissue differentiation and maintenance (Figure 16C). GO biological process enrichment analysis demonstrated strong associations with intermediate filament cytoskeleton organization, intermediate filament organization, and lens development in the camera-type eye, suggesting that BFSP1 and its network contribute to maintaining cellular architecture and mechanical stability within specialized tissues, such as the eye lens (Figure 16D). GO cellular component analysis revealed enrichment of BFSP1-interacting proteins in cardiac myofibrils, intermediate filaments, and supramolecular fibers (Figure 16E), indicating broader functional roles for BFSP1 in structural integrity and contractile apparatuses beyond ocular tissues, potentially implicating BFSP1 in muscle and cytoskeletal biology. GO molecular function analysis identified a significant association between the structural constituents of the eye lens, highlighting the essential contribution of BFSP1 and its partners to lens transparency, refractive properties, and overall visual function (Figure 16F). Disease gene enrichment analysis demonstrated that the BFSP1 PPI network is significantly associated with Alexander’s disease, cataracts, various eye diseases, and nervous system disorders. This finding suggests that dysregulation of BFSP1 and its interacting proteins contributes to the pathogenesis of diverse neurodegenerative and ocular diseases, providing potential avenues for biomarker discovery and therapeutic interventions (Figure 16G). Collectively, these results indicate that the BFSP1-associated PPI network plays a critical role in the structural maintenance of specialized tissues, such as the eye lens, with broader implications for disease processes that affect the nervous system and ocular health.

### 3.11. Protein Expression and Cell Migration in HepG2 Cells

**Figure 17:**
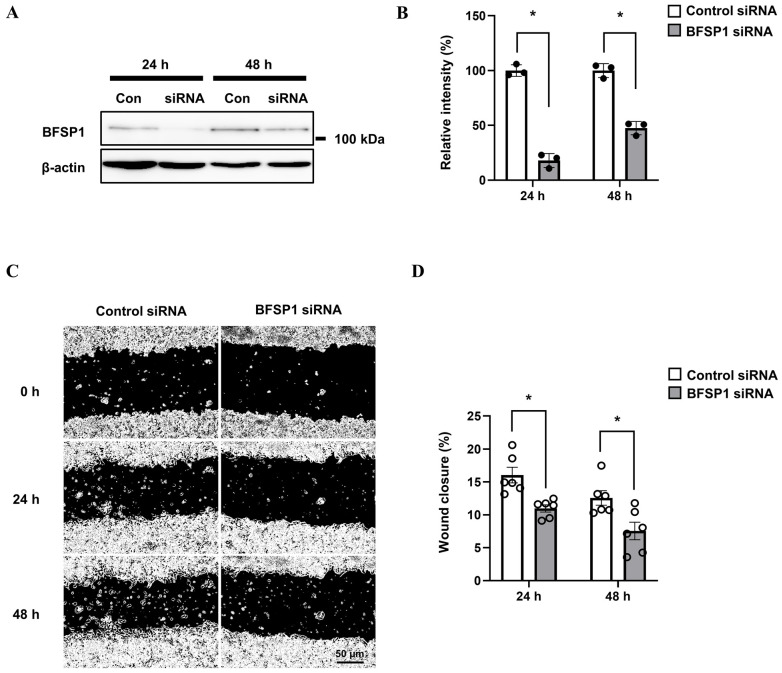
Effects of BFSP1 knockdown on cell migration in HepG2 cells. (**A**) Western blot analysis showing reduced BFSP1 protein expression in cells transfected with BFSP1-specific siRNA compared to control siRNA at 24 and 48 h. β-Actin was used as a loading control. (**B**) Densitometric quantification of BFSP1 protein levels normalized to β-actin. Data are presented as the mean ± SEM (n = 3 per group). Statistical analysis was performed using ANOVA with Tukey’s post hoc test (* *p* < 0.05 vs. control siRNA). Prior to ANOVA, normality was confirmed using the Shapiro–Wilk test. (**C**) Representative images of wound healing assays at 0, 24, and 48 h after scratch in control and BFSP1siRNA-transfected HepG2 cells. (**D**) Quantitative analysis of wound closure percentage at 24 and 48 h post-scratch. Data are shown as the mean ± SEM (n = 6 per group). Statistical analysis was performed using two-way ANOVA followed by Tukey’s post hoc test (* *p* < 0.01 vs. control siRNA). Prior to ANOVA, normality was confirmed using the Shapiro–Wilk test.

To examine the functional role of BFSP1 in liver cancer cells, HepG2 cells were transfected with either control short interfering RNA (siRNA) or BFSP1-targeting siRNA (si-BFSP1). Western blot analysis demonstrated a significant reduction in BFSP1 protein expression in si-BFSP1-transfected cells compared with controls at 24 and 48 h post-transfection (Figure 17A,B). Specifically, the mean expression of BFSP1 was reduced by 82% at 24 h and by 53% at 48 h post-transfection relative to control. To assess the effect of BFSP1 knockdown on cell motility, a wound healing assay was conducted. Cells transfected with si-BFSP1 exhibited a markedly decreased wound closure rate at 24 and 48 h relative to control cells (Figure 17C,D). These results suggest that BFSP1 expression is essential for maintaining the migratory capacity of HepG2 cells, suggesting that BFSP1 plays a critical role in the motility and metastatic potential of hepatocellular carcinoma cells.

## 4. Discussion

LIHC continues to represent a significant global health burden, accounting for more than 90% of primary liver cancers and consistently ranking among the leading causes of cancer-related mortality worldwide [1,3,4]. This malignancy primarily develops in individuals with chronic liver conditions, especially cirrhosis, and is strongly associated with risk factors such as chronic hepatitis B and C infections, alcohol-related liver disease, non-alcoholic fatty liver disease, and exposure to environmental carcinogens [5,6,7]. Despite advances in diagnostic and therapeutic approaches, the prognosis of LIHC remains poor, with 5-year survival rates below 20% [8,9]. Poor outcomes are largely due to late-stage diagnosis, pronounced tumor heterogeneity, frequent recurrence, early metastasis, and emergence of drug resistance [10,11,71,72].

Traditional prognostic models based on clinical and pathological parameters insufficiently reflect the biological and molecular complexities of LIHC, thereby limiting their effectiveness in personalized management [12]. Recent advances in high-throughput multi-omics technologies have enabled the identification of genetic, epigenetic, and immune-related biomarkers, facilitating the development of more robust prognostic models and precise oncology approaches [13,14,15]. In this context, cytoskeletal proteins, particularly intermediate filaments, have been shown to contribute to tumor pathogenesis and cancer progression [18,20].

BFSP1 has traditionally been regarded as a lens-specific structural protein; however, recent pan-cancer analyses have revealed its aberrant expression in several malignancies, including LIHC [19]. In this study, we comprehensively investigated the functional and clinical significance of BFSP1 in liver cancer and provided direct evidence that BFSP1 upregulation correlates with aggressive tumor biology and poor prognosis [21]. These findings are consistent with previous studies demonstrating the prognostic and diagnostic utility of DNA methylation signatures in LIHC [34,48], further suggesting that BFSP1 expression is susceptible to epigenetic deregulation. Moreover, the observed associations between BFSP1 and TIICs highlight the critical role of the tumor immune microenvironment in influencing LIHC outcomes and therapeutic responses [25,27]. Our data reinforce and extend recent evidence advocating clinical integration of molecular biomarkers and drug sensitivity profiles to advance precision oncology approaches [74].

Integrative analysis showed that BFSP1 exerts a multifaceted oncogenic role in LIHC. We found that BFSP1 was significantly upregulated at the mRNA level in both LIHC tissues and cell lines, with elevated expression correlating with advanced clinicopathological characteristics and a significantly poorer prognosis. Dysregulation of BFSP1 was associated with promoter hypomethylation and CNVs, indicating its susceptibility to both epigenetic and genomic alterations. These findings raise the possibility that BFSP1 promoter methylation could be leveraged as a biomarker for clinical risk stratification. Furthermore, in vitro experiments demonstrated that the functional loss of BFSP1, achieved by siBFSP1 transfection, resulted in a marked reduction in migratory activity. This finding suggests that BFSP1 enhances malignant phenotypes by promoting cell motility, thereby supporting its proposed role in driving tumor aggressiveness. In addition, we demonstrated that BFSP1 expression correlates with TIICs, indicating its potential role in shaping an immunosuppressive microenvironment and influencing responsiveness to immunotherapy. Moreover, drug sensitivity analyses revealed that elevated BFSP1 expression modulates the response to multiple anticancer agents, underscoring its potential as a predictive biomarker for therapeutic stratification.

Nonetheless, this investigation has several limitations. Our analysis relied primarily on retrospective in silico analyses of the single, publicly available TCGA LIHC datasets, which inherently limits the generalizability of the findings and necessitates cautious interpretation. Especially, the observed statistical associations are subject to dataset biases related to the specific patient demographics, treatment protocols, and sample processing methodologies across contributing centers. Furthermore, the TCGA cohort shows considerable clinical and molecular heterogeneity which may introduce confounding effects on the observed associations with BFSP1. Because the data are observational, causal relationships cannot be firmly determined, and revers causality cannot be fully excluded—advanced tumor characteristics or subsequent treatment may affect BFSP1 expression rather than BSFP1 influencing disease progression.

From a methodological standpoint, our study is limited by the lack of validation in an independent ethnically diver cohort and the absence of extensive in vivo experiments. Functional validation was restricted to a single cell line (HepG2), and no in vivo experiments were performed ton confirm the in vitro results. The predictive value of BFSP1 for drug sensitivity requires further validation in prospective clinically annotated cohorts and controlled clinical trials. Moreover, the mechanism of BFSP1’s regulatory effects on immune cell infiltration, methylation, and metabolism should be determined in comprehensive molecular and translational investigations. To advance the clinical translation of these findings, future studies should validate BFSP1 as a prognostic and predictive biomarker in larger, independent ethnically diverse LIHC cohorts. Future studies should employ in vivo models, such as patient-derived xenografts, to elucidate the functional role of BFSP1 in tumorigenesis, progression, and metastasis. It is also essential to evaluate the molecular mechanisms by which BFSP1 modulates evasion and resistance to therapy. Furthermore, pharmacological targeting of BFSP1, including combination regimens with systemic or immune-based therapies, should be explored. Finally, expanding multi-omics and single-cell analyses will be critical to comprehensively define the role of BFSP1 in broader regulatory networks.

## 5. Conclusions

We identified BFSP1 as a multifaceted biomarker in LIHC, impacting tumor progression, immune microenvironment, and therapeutic response. These findings provide a foundation for further investigation of BFSP1 as a potential clinical target in precision oncology.

## Figures and Tables

**Table 1:** Clinicopathological characteristics of BFSP1 expression using Kaplan–Meier database in LIHC.

Clinicopathological Characteristics	Overall Survival			Relapse-Free Survival			Progression-Free Survival			Disease-Specific Survival		
	(*n* = 364)			(*n* = 316)			(*n* = 370)			(*n* = 362)		
	*N*	Hazard Ratio	*p*-Value	*N*	Hazard Ratio	*p*-Value	*N*	Hazard Ratio	*p*-Value	*N*	Hazard Ratio	*p*-Value
**Sex**												
Male	246	1.49 (0.95–2.33)	0.08	210	1.47 (0.98–2.18)	0.059	249	1.65 (1.15–2.37)	**0.0059**	244	1.92 (1.07–3.45)	**0.027**
Female	118	1.11 (0.63–1.96)	0.71	106	1.82 (1–3.33)	**0.048**	121	1.96 (1.16–3.3)	**0.01**	118	1.02 (0.49–2.1)	0.96
**Stage**												
I	170	1.39 (0.75–2.56)	0.29	153	1.65 (0.95–2.84)	0.071	171	1.7 (1.03–2.81)	**0.036**	168	2.04 (0.82–5.1)	0.12
I + II	253	1.39 (0.85–2.26)	0.19	228	1.52 (1–2.31)	0.05	256	1.79 (1.22–2.63)	**0.0026**	251	2.12 (1.02–4.39)	**0.04**
II	83	1.54 (0.69–3.42)	0.29	75	1.22 (0.62–2.4)	0.56	85	1.71 (0.93–3.15)	0.083	83	3.33 (0.92–12.13)	0.052
II + III	166	1.51 (0.94–2.44)	0.087	145	1.48 (0.95–2.32)	0.084	170	1.57 (1.05–2.34)	**0.027**	166	2.09 (1.12–3.91)	**0.018**
III	83	1.85 (1.02–3.36)	**0.039**	70	1.37 (0.75–2.51)	0.3	85	1.82 (1.05–3.16)	**0.029**	83	2.22 (1.08–4.59)	0.027
III + IV	87	1.82 (1.03–3.23)	**0.037**	70	1.37 (0.75–2.51)	0.3	90	1.81 (1.06–3.07)	**0.027**	87	2.01 (1–4.02)	0.044
IV	4	-	-	0	-	-	5	-	-	3	-	-
**Grade**												
I	55	1.63 (0.64–4.15)	0.3	45	2.16 (0.81–5.72)	0.11	55	1.73 (0.79–3.77)	0.17	55	0.75 (0.21–2.59)	0.64
II	174	1.55 (0.92–2.61)	0.099	149	1.68 (1.03–2.74)	**0.036**	177	1.95 (1.25–3.02)	**0.0025**	171	2.27 (1.13–4.58)	**0.019**
III	118	1.48 (0.81–2.74)	0.2	107	1.29 (0.75–2.21)	0.35	121	1.67 (1–2.78)	**0.048**	119	1.99 (0.89–4.44)	0.086
IV	12	-	-	11	-	-	12	-	-	12	-	-
**AJCC_T**												
I	180	1.67 (0.92–3.02)	0.087	160	1.74 (1.02–2.97)	**0.039**	181	1.84 (1.13–3)	**0.013**	178	2.11 (0.91–4.89)	0.074
II	90	1.65 (0.78–3.5)	0.19	80	1.16 (0.61–2.19)	0.65	93	1.75 (0.99–3.08)	**0.05**	91	2.4 (0.84–6.82)	0.09
III	78	1.37 (0.75–2.5)	0.3	67	1.35 (0.72–2.53)	0.34	80	1.65 (0.93–2.91)	0.081	77	1.6 (0.76–3.35)	0.21
IV	13	-	-	6	-	-	13	-	-	13	-	-
**Vascular invasion**												
None	203	1.7 (1–2.89)	**0.047**	175	1.57 (0.97–2.55)	0.064	205	1.68 (1.07–2.64)	**0.022**	201	2.27 (1.05–4.88)	**0.032**
Micro	90	1.03 (0.47–2.22)	0.95	82	1.32 (0.7–2.49)	0.4	92	1.49 (0.84–2.65)	0.17	90	1.17 (0.39–3.5)	0.77
Macro	16	-	-	14	-	-	16	-	-	14	-	-
**Race**												
White	181	1.2 (0.75–1.91)	0.45	147	1.65 (1.05–2.61)	**0.029**	184	1.84 (1.24–2.73)	**0.0023**	179	1.12 (0.64–1.98)	0.69
Asian	155	3.09 (1.62–5.92)	**0.00033**	145	1.33 (0.8–2.21)	0.27	157	1.55 (0.96–2.48)	0.068	154	4.31 (1.72–10.82)	**0.00068**
**Alcohol consumption**												
Yes	115	1.34 (0.72–2.5)	0.36	99	1.23 (0.69–2.21)	0.48	117	1.53 (0.91–2.56)	0.11	117	1.24 (0.6–2.57)	0.56
None	202	1.5 (0.94–2.38)	0.084	183	1.78 (1.13–2.79)	**0.011**	205	1.94 (1.29–2.92)	**0.0012**	199	2.5 (1.29–4.84)	**0.0048**
**Hepatitis virus**												
Yes	150	1.99 (1.02–3.86)	**0.039**	139	1.13 (0.69–1.86)	0.62	153	1.32 (0.83–2.09)	0.24	151	2.9 (1.19–7.07)	**0.014**
None	167	1.17 (0.74–1.85)	0.49	143	1.7 (1.03–2.81)	**0.036**	169	1.96 (1.26–3.05)	**0.0022**	165	1.12 (0.62–2)	0.71

**Table 2:** Prognostic significance of BFSP1 expression in using PrognoScan database in various cancer types.

Dataset	Cancer Type	Endpoint	*N*	In (HRhigh/HRlow)	Cox *p*-Value	HR (95% Cllow—Clup)
GSE13507	Bladder cancer	Disease-specific survival	165	1.05	0.002101	4.48 (1.72–11.66)
GSE12276	Breast cancer	Relapse-free survival	204	0.65	0.004341	1.26 (1.08–1.48)
GSE31210	Lung cancer	Relapse-free survival	204	−1.85	0.019986	0.76 (0.60–0.96)
GSE4475	Blood cancer	Overall survival	158	0.77	0.020094	7.65 (1.38–42.49)
GSE1379	Breast cancer	Relapse-free survival	60	2.1	0.024794	1.83 (1.08–3.10)
GSE1378	Breast cancer	Relapse-free survival	60	1.31	0.031161	1.51 (1.04–2.18)
GSE8970	Blood cancer	Overall survival	34	1.28	0.046263	1.80 (1.01–3.22)
E-TABM-158	Breast cancer	Relapse-free survival	117	−1.72	0.04948	0.11 (0.01–1.00)

**Table 3:** BFSP1- associated DNA methylation probes in LIHC.

Gene	Probe	Chr	Position	Average of Cancer Sample	Average of Normal Sample	*p*-Value
*BFSP1*	cg27500292	chr20	17525532	0.62	0.78	<0.001
	cg06521030	chr20	17528778	0.89	0.93	<0.001
	cg00412842	chr20	17530834	0.09	0.18	<0.001
	cg07217075	chr20	17531181	0.02	0.01	0.01
	cg07904983	chr20	17531343	0.02	0.02	0.02
	cg26371206	chr20	17531563	0.1	0.17	<0.001
	cg04346572	chr20	17531649	0.08	0.19	<0.001
	cg23985077	chr20	17532007	0.72	0.89	<0.001
	cg23032598	chr20	17532150	0.76	0.85	<0.001
	cg02795691	chr20	17533752	0.52	0.68	<0.001
	cg18815565	chr20	17559363	0.81	0.92	<0.001
	cg04264633	chr20	17559916	0.42	0.49	<0.001
	cg14801864	chr20	17560330	0.68	0.8	<0.001
	cg22276571	chr20	17568954	0.15	0.21	<0.001
	cg10573932	chr20	17569052	0.13	0.16	0.008
	cg23651728	chr20	17530765	0.1	0.1	0.6
	cg04263118	chr20	17531532	0.05	0.06	0.3
	cg14158573	chr20	17569037	0.15	0.15	0.9
	cg19765886	chr20	17569220	0.02	0.02	0.07
	cg03925157	chr20	17569310	0.13	0.14	0.08
	cg00473384	chr20	17569793	0.02	0.03	0.08
	cg11320690	chr20	17569829	0.02	0.02	0.3
	cg12878213	chr20	17569836	0.04	0.04	0.2
	cg06260159	chr20	17569935	0.04	0.04	0.8
	cg01993865	chr20	17570045	0.02	0.02	1
	cg13462232	chr20	17570521	0.05	0.05	0.5

**Table 4:** Correlation between DNA methylation probes of the BFSP1 and histological grade in LIHC.

Gene	Probe	Chr	Position	G1 Beta Average	G2 Beta Average	G3 Beta Average	G4 Beta Average	*p*-Value
*BFSP1*	cg27500292	chr20	17525532	0.64	0.63	0.6	0.55	0.0005
	cg06521030	chr20	17528778	0.89	0.89	0.89	0.87	
	cg23651728	chr20	17530765	0.08	0.1	0.09	0.07	
	cg00412842	chr20	17530834	0.1	0.1	0.07	0.06	
	cg07217075	chr20	17531181	0.04	0.01	0.02	0.01	
	cg07904983	chr20	17531343	0.03	0.02	0.02	0.02	
	cg04263118	chr20	17531532	0.06	0.05	0.06	0.04	
	cg26371206	chr20	17531563	0.1	0.09	0.09	0.08	
	cg04346572	chr20	17531649	0.1	0.08	0.08	0.07	
	cg23985077	chr20	17532007	0.8	0.72	0.7	0.59	
	cg23032598	chr20	17532150	0.8	0.78	0.73	0.65	
	cg02795691	chr20	17533752	0.63	0.53	0.47	0.37	
	cg18815565	chr20	17559363	0.8	0.8	0.82	0.8	
	cg04264633	chr20	17559916	0.42	0.41	0.43	0.45	
	cg14801864	chr20	17560330	0.68	0.69	0.68	0.64	
	cg22276571	chr20	17568954	0.15	0.15	0.13	0.11	
	cg14158573	chr20	17569037	0.16	0.16	0.14	0.11	
	cg10573932	chr20	17569052	0.14	0.14	0.12	0.12	
	cg19765886	chr20	17569220	0.02	0.02	0.02	0.02	
	cg03925157	chr20	17569310	0.13	0.13	0.13	0.12	
	cg00473384	chr20	17569793	0.02	0.02	0.02	0.02	
	cg11320690	chr20	17569829	0.02	0.02	0.02	0.02	
	cg12878213	chr20	17569836	0.04	0.04	0.04	0.03	
	cg06260159	chr20	17569935	0.04	0.04	0.04	0.05	
	cg01993865	chr20	17570045	0.02	0.02	0.02	0.01	
	cg13462232	chr20	17570521	0.05	0.04	0.05	0.04	

**Table 5:** Correlation between BFSP1 expression and TIICs.

Cancer	Gene	Type	Cell Type	R	*p*-Value
LIHC	BFSP1	Expression	Bcell	0.46	<0.001
			nTreg	0.21	<0.001
			Tr1	0.21	<0.001
			CD8-T	0.16	<0.001
			CD8-naïve	0.16	<0.001
			NKT	0.15	<0.001
			MAIT	−0.33	<0.001
			Macrophage	−0.29	<0.001
			Monocyte	−0.25	<0.001
			Tfh	−0.2	<0.001
			CD4_naïve	−0.19	<0.001
			Th17	−0.17	<0.001
			NK	−0.16	<0.001
		CNV	Bcell	0.18	<0.001
			nTreg	0.13	0.01
			CD8_T	0.14	0.006
			Th1	0.12	0.02
			CD4_naïve	−0.18	0
			MAIT	−0.17	<0.001
			Th17	−0.13	0.02
			Monocyte	−0.13	0.02
		Methylation	Tfh	0.31	<0.001
			CD4_T	0.28	<0.001
			MAIT	0.25	<0.001
			NK	0.22	<0.001
			Th1	0.18	<0.001
			iTreg	0.14	0.008
			Macrophage	0.13	0.01
			CD8_T	0.13	0.01
			NKT	0.13	0.01
			Neutrophil	−0.27	<0.001
			Bcell	−0.27	<0.001
			Tr1	−0.15	0.003
			Th17	−0.11	0.03

**Table 6:** Interactions between BFSP1 expression and chemicals in LIHC using the CTD.

Chemical Name	ID	Interaction Actions
2,3′,4,4′,5-Pentachlorobiphenyl	C070055	Increases expression
2,3,5-Trichloro-6-phenyl-(1,4)benzoquinone	C587734	Increases expression
Acetamide	C030686	Increases expression
Afuresertib	C000593263	Increases expression
Aristolochic acid	C000228	Increases expression
Benzo(b)fluoranthene	C006703	Increases expression
Copper sulfate	D019327	Increases expression
ethyl-p-hydroxybenzoate	C012313	Increases expression
Fenvalerate	C017690	Increases expression
Folic acid	D005492	Increases expression
Gentamicins	D005839	Increases expression
Hexachlorocyclohexane	D001556	Increases expression
(+)-JQ1 compound	C561695	Increases expression
Lipopolysaccharides	D008070	Increases expression
Methamphetamine	D008694	Increases expression
Particulate matter	D052638	Increases expression
Propylthiouracil	D011441	Increases expression
*S*-(1,2-Dichlorovinyl)cysteine	C039961	Increases expression
*S*-(1,2-Dichlorovinyl)cysteine	C039961	Increases expression
Sodium arsenite	C017947	Increases expression
Tetrachlorodibenzodioxin	D013749	Increases expression
Thioacetamide	D013853	Increases expression
Trichloroethylene	D014241	Increases expression
Triptolide	C001899	Increases expression
Triptonide	C084079	Increases expression
Vinclozolin	C025643	Increases expression
Acetaminophen	D000082	Decreases expression
Acrylamide	D020106	Decreases expression
Ammonium 2,3,3,3-tetrafluoro-2- (heptafluoropropoxy)-propanoate	C000611729	Decreases expression
Belinostat	C487081	Decreases expression
Bilirubin	D001663	Decreases expression
bisphenol A	C006780	Decreases expression
Dichlorodiphenyl dichloroethylene	D003633	Decreases expression
Dietary fats	D004041	Decreases expression
Dinophysistoxin 1	C051904	Decreases expression
Domoic acid	C012301	Decreases expression
ICG 001	C492448	Decreases expression
Sunitinib	D000077210	Decreases expression
Testosterone	D013739	Decreases expression
Thalidomide	D013792	Decreases expression
Tobacco smoke pollution	D014028	Decreases expression
Tretinoin	D014212	Decreases expression
Zinc sulfate	D019287	Decreases expression
Zoledronic acid	D000077211	Decreases expression

**Table 7:** Interactions between BFSP1-related genes and chemicals in LIHC using CTD.

Gene	Similarity Index	Common Interaction Chemicals
*CCDC88B*	0.2784	27
*CCDC102A*	0.2747	25
*OLFM2*	0.2727	24
*SYTL3*	0.2708	26
*CPNE5*	0.2673	27
*LYL1*	0.266	25
*MGAM*	0.2655	30
*PRX*	0.2626	26
*SHANK1*	0.2621	27
*KCTD13*	0.2604	25
*PLEKHA4*	0.26	26
*ANKS6*	0.2558	22
*SH3RF2*	0.2547	27
*KCNAB1*	0.2542	30
*KCNH4*	0.2533	19
*CASS4*	0.2529	22
*C5AR2*	0.2526	24
*MPDU1*	0.2526	24
*ARHGAP27*	0.2524	26
*NPTXR*	0.2524	26

## Data Availability

All data are available upon reasonable request from the corresponding author.

## Abbreviations

The following abbreviations are used in this manuscript:

BFSP1	beaded filament structural protein 1
CNV	copy number variation
CTD	Comparative Toxicogenomics Database
DSS	disease-specific survival
GO	Gene Ontology
GSCA	Gene Set Context Analysis
KEGG	Kyoto Encyclopedia of Genes and Genomes
LIHC	liver hepatocellular carcinoma
HR	hazard ratio
MAIT	mucosal-associated invariant T
NK	natural killer
OS	overall survival
PPI	protein–protein interaction
TCGA	The Cancer Genome Atlas
Treg	regulatory T cell
